# Impact of Positional Isomerism on Pathway Complexity in Aqueous Media

**DOI:** 10.1002/anie.201911531

**Published:** 2020-02-03

**Authors:** Ingo Helmers, Bowen Shen, Kalathil K. Kartha, Rodrigo Q. Albuquerque, Myongsoo Lee, Gustavo Fernández

**Affiliations:** ^1^ Organisch-Chemisches Institut Westfälische Wilhelms-Universität Münster Corrensstraße 40 48149 Münster Germany; ^2^ State Key Lab of Supramolecular Structure and Materials College of Chemistry Jilin University Changchun 130012 China

**Keywords:** amphiphilic systems, aqueous self-assembly, BODIPY dyes, cooperativity, pathway complexity

## Abstract

Pathway complexity has become an important topic in recent years due to its relevance in the optimization of molecular assembly processes, which typically require precise sample preparation protocols. Alternatively, competing aggregation pathways can be controlled by molecular design, which primarily rely on geometrical changes of the building blocks. However, understanding how to control pathway complexity by molecular design remains elusive and new approaches are needed. Herein, we exploit positional isomerism as a new molecular design strategy for pathway control in aqueous self‐assembly. We compare the self‐assembly of two carboxyl‐functionalized amphiphilic BODIPY dyes that solely differ in the relative position of functional groups. Placement of the carboxyl group at the 2‐position enables efficient pairwise H‐bonding interactions into a single thermodynamic species, whereas *meso*‐substitution induces pathway complexity due to competing hydrophobic and hydrogen bonding interactions. Our results show the importance of positional engineering for pathway control in aqueous self‐assembly.

## Graphical abstract

**A matter of position**: Rational placement of functional groups for weak interactions has been exploited as a new method for pathway control in aqueous self‐assembly. The results may contribute to understanding the molecular design principles that govern the outcome of self‐assembly processes in water.

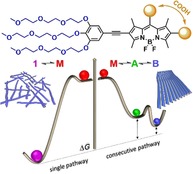

## Introduction

Biomolecular systems are able to adapt their morphology in aqueous media to different environmental conditions.^1^ A particularly illustrative example is the α‐protein tropomyosin, which can exist in eight distinct types of aggregates.^2^ This unparalleled pathway control has motivated the investigation of these phenomena in synthetic self‐assembled counterparts. In recent years, pathway complexity^3^ has been observed for different types of building blocks both in non‐polar^4^ and aqueous media.^5^ Control over the competing aggregation pathways in these molecular systems is typically achieved by optimization of sample preparation protocols. These processes are generally tedious and often require a very specific set of experimental conditions (that is, concentration, temperature, solvent composition, and others).^6^ Alternatively, these complex sample preparation methods could be simplified by molecular design strategies, which have been recently introduced to broaden the scope of pathway complexity. To date, these approaches have mainly focused on the geometrical modification of the building blocks either by systematic size variation of substituents4d, 4h or length variation of a given molecular fragment (for example, π‐system,4g, 5m alkyl spacers,4e, 5d or side chains5a, 5j). In this regard, controlling pathway complexity in aqueous media by molecular design is particularly challenging, as the competition between hydrophobic and other non‐covalent interactions5b–5f, 5h, 5i, 5k, 5l makes the self‐assembly considerably less predictable. In amphiphilic self‐assembly, fine‐tuning of the hydrophilic/hydrophobic ratio is a well‐known strategy for morphology control^7^ and, to a minor extent, has also been observed to induce pathway complexity.5a, 5j, 5m However, understanding the molecular design principles that govern the existence of pathway complexity in self‐assembly still remains an open question and new approaches are required.

**Scheme 1:**
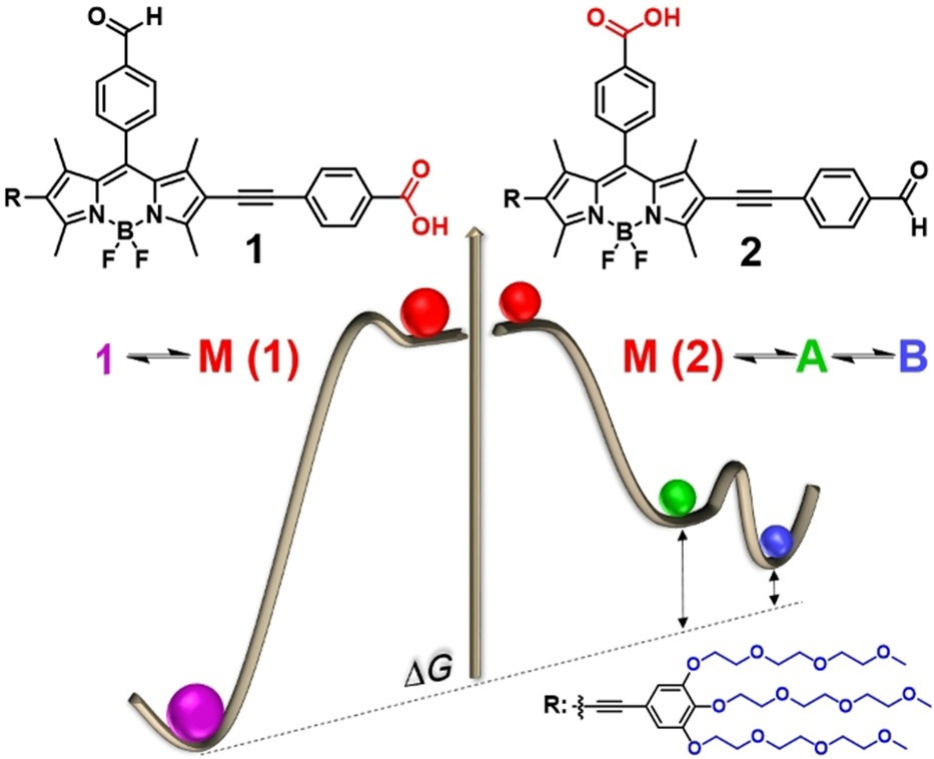
Molecular structures of **1** and **2** and corresponding energy profiles illustrating their aggregation pathways.

In the present manuscript, we introduce the concept of positional isomerism as a new molecular design strategy for pathway selection in aqueous self‐assembly. To this end, we have synthesized two positional isomers of an amphiphilic BODIPY dye that only differ in the location of the functional groups (compounds **1** and **2** in Scheme 1; for synthesis and characterization, see the Supporting Information). In addition to the typical methyl substitution of the BODIPY core,^8^ both dyes are functionalized at the 6‐position with an ethynylbenzene moiety bearing three hydrophilic triethylene glycol (TEG) chains to provide water solubility. Additionally, the choice of a carboxylic acid group at either the 2‐ (**1**) or the *meso*‐position (**2**) of the BODIPY core was made based on its potential to establish hydrogen bonds both in non‐polar solvents^9^ and water when arranged in a local hydrophobic environment.5d, 10 Finally, the presence of a bulky aromatic electron‐withdrawing substituent at the BODIPY *meso*‐position is expected to provide the system with enhanced dynamics by hindering a face‐to‐face stacking into non‐emissive H‐type aggregates.^11^, ^12^

The location of the carboxylic acid is observed to determine pathway complexity: if this group is placed on the opposite side of the hydrophilic chains, pairwise hydrogen bonding of the carboxyl groups can efficiently occur in a hydrophobic microenvironment, leading to thermodynamically controlled lamellar nanostructures. In contrast, the location of the acid at the *meso*‐position induces competition of entropic and enthalpic contributions: hydrophobic and aromatic interactions initially drive the self‐assembly into metastable unordered nanostructures. Over time, steric effects induce a molecular rearrangement into thermodynamically controlled flexible J‐type^13^ nanofibers that are stabilized by partial hydrogen bonding of the carboxylic acids.

## Results and Discussion

**Figure 1:**
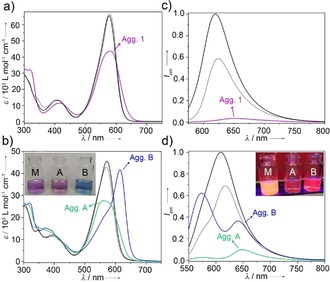
a), b) UV/Vis absorption and c), d) normalized emission spectra of compounds **1** (a,c) and **2** (b,d) in different solvents (*c=*20 μm) at 298 K. *λ*_exc_=530 nm. Insets (b,d): monomer (M, left); aggregate **A** (center); aggregate **B** (right). The black and gray plots in (a–d) correspond to the monomeric spectra in 1,2‐propanediol and DCM, respectively. The spectra of aggregates **1**, **A**, and **B** in water are shown in violet, green, and blue, respectively.

First insights into the self‐assembly of **1** and **2** were gained from solvent‐dependent absorption studies (*c=*20 μm, 298 K). Compound **1** exists in a monomeric state (M) in both polar and non‐polar organic solvents, showing absorption maxima at about 577 nm (S_0_→S_1_ transition of the BODIPY) along with a low‐intensity transition (S_0_→S_2_) at about 406 nm (Figure S11, Supporting Information; for selected “good” solvents such as dichloromethane (DCM) and 1,2‐propanediol, see Figure 1 a). Similar spectra with characteristic BODIPY bands at 574 nm and 409 nm obtained for derivative **2** (Figure 1 b) show that the different substitution pattern negligibly influences the spectral changes in the molecularly dissolved state. This is further supported by the presence of sharp emission bands with maxima at comparable wavelengths (*λ*≈620 nm) for both molecules in a variety of organic solvents (Figures 1 c,d and S11, Table S1).

This behavior changes considerably if the systems are investigated in water (pH = 7). For **1**, the concomitant decrease in absorption and spectral broadening compared with the spectra recorded in organic solvents are indicative of aggregation (Figure 1 a). The negligible shift of the absorption maximum during this process suggests that an ideal face‐to‐face H‐type stacking of the BODIPY dyes^14^ may possibly be hindered by the sterically demanding benzaldehyde group at the BODIPY *meso*‐position. Emission studies show a red‐shift and drastic quenching of the fluorescence (Figure 1 c).

**Scheme 2:**
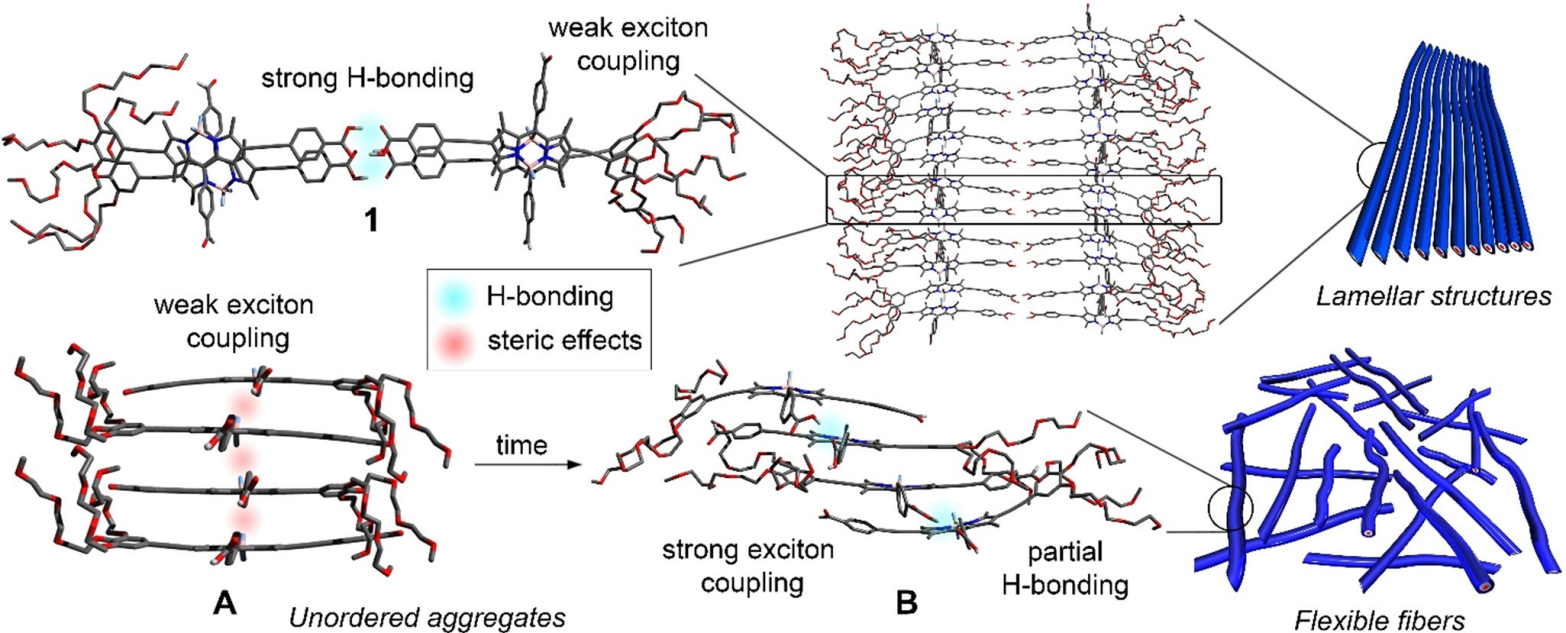
Schematic representation of the self‐assembly of **1**, **A**, and **B**. The tetramer stacks of **1** and **B** are PM6‐optimized structures.

For **2**, UV/Vis absorption and emission studies of a freshly prepared solution in water show a trend very similar to **1** (green spectra in Figure 1 b,d), indicating weakly coupled dye units in the assembled structure (denoted as aggregate **A** in the following). Surprisingly, upon aging for a minimum of 20 h or after thermal annealing, aggregate **A** transforms into a new, energetically more favorable assembly **B** with strong J‐type exciton coupling (Δ*λ*=40 nm, *λ*_max_=615 nm; Figure 1 b, blue spectrum), which is accompanied by a color change from violet to blue (Figure 1 b, inset). This aggregate species **B** displays higher emission intensity than **A** [fluorescence quantum yield, *ϕ*_F_(**B**)=6.4 % vs. *ϕ*_F_(**A**)=2.4 %], in accordance with a plausible J‐type aggregate formation. The additional blue‐shifted emission band at 570 nm may be attributed to the existence of defects in the packing of **B** (Scheme 2), which is supported by different excitation spectra upon collecting emission at 570 or 650 nm (Figure S12).

**Figure 2:**
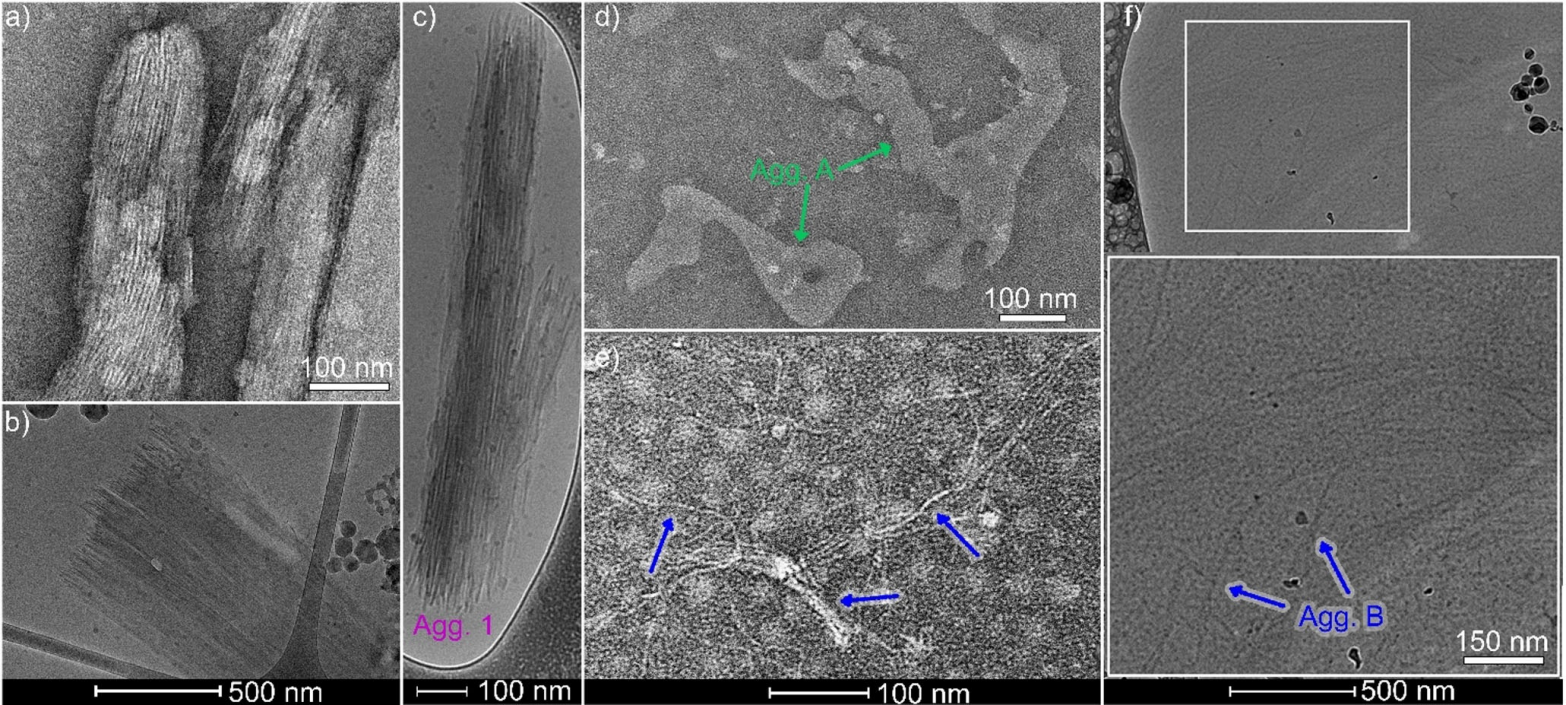
a) TEM and b), c) cryo‐TEM images of **1**. d) TEM image of **A**. e) TEM and f) cryo‐TEM images of **B**. Inset in (f): zoomed‐in cryo‐TEM image. For all studies, the compound was dissolved in water at *c*=20 μm and placed on a carbon‐coated copper grid. Negative staining has been used for TEM.

This different propensity of **1** and **2** to undergo pathway complexity prompted us to investigate the nanoscale morphology and size of **1**, **A**, and **B** by cryogenic transmission electron microscopy (cryo‐TEM), TEM, and dynamic light scattering (DLS). As shown by TEM and cryo‐TEM, compound **1** self‐assembles into rigid fibrils with lengths of ≈800 nm and a uniform diameter of approximately 5 nm (Figures 2 a–c and S13), which closely matches twice the molecular length. The images also reveal that the individual straight fibrils have a strong tendency to bundle into lamellar structures. These results can be explained by the formation of extended stacks of hydrogen‐bonded dimers of **1** (via the carboxylic acid).5d Within this arrangement, the polar TEG chains would be exposed to the aqueous medium, thus enabling the bundling of these fibrils by strong lateral association of the TEG chains, as also observed for other amphiphilic molecules.^15^, ^16^ The anisotropy of these structures is further supported by the observation of angular‐dependent hydrodynamic radii in DLS studies (Figure S14).^17^

On the contrary, the assemblies of **A** are identified by TEM as anisotropic nanostructures with ill‐defined shape (Figure 2 d). Aging this solution leads to the formation of flexible fibres (**B**) with lengths of ≈900 nm and a diameter of 5 nm (Figures 2 e,f and S13). Compared to **1**, these fibers are more flexible and have a lower tendency to bundle. The changes in size and morphology for the transformation of **A** to **B** were also monitored by DLS studies (Figure S14).

**Figure 3:**
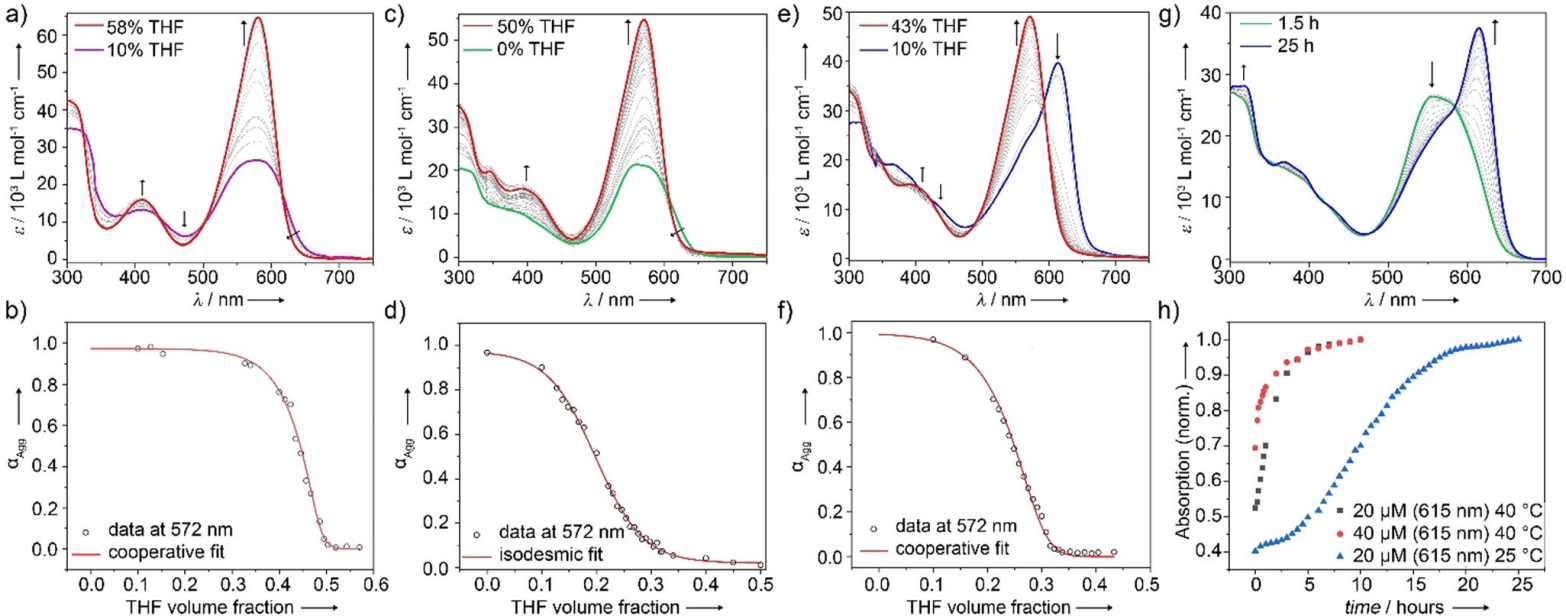
UV/Vis studies at different THF/water ratios at *c=*20 μm and RT for a) **1**, c) **A**, and e) **B**. Plot of *α*_agg_ vs. volume fraction of THF monitored at *λ*=572 nm for b) **1**, d) **A**, and f) **B** along with corresponding fits to the denaturation model. g) Time‐dependent evolution of assembly **A** into **B** in H_2_O (*c=*20 μm) at room temperature. h) Absorbance vs. time at *λ*=615 nm at different concentrations and temperatures.

To gain mechanistic insights into these different aggregation processes, spectroscopic studies using different ratios of good/poor solvent (THF/water) were carried out at various concentrations (8–40 μm, Figures 3 and S15–S17). For this purpose, the disassembly of aqueous aggregates of **1**, **A**, and **B** was monitored by the addition of increasing amounts of the respective monomer solutions in THF. Addition of aliquots of monomeric **1** in THF to an aggregate solution of **1** in water at 20 μm initiated the disassembly process, which was complete when the volume fraction of THF in water exceeded 50 % (Figures 3 a, S15). The non‐sigmoidal plot of the fraction of aggregated species (*α*_agg_) vs. the volume fraction of THF monitored at 572 nm indicates a cooperative process. Subsequent fit to the denaturation model^18^ (Figure 3 b) yields an average Gibbs energy (Δ*G*°) of −52.4 kJ mol^−1^ (Table S2).

For compound **2**, full denaturation of the kinetically controlled species **A** requires a lower volume fraction of THF (≈30 %) than **1** at the same concentration (Figures 3 c and S16), indicating a higher stability of the latter aggregate. Additionally, the disassembly process of **A** is described by an isodesmic mechanism (Figure 3 d), yielding a Δ*G*° of −44.3 kJ mol^−1^. In contrast, **B** follows a cooperative mechanism (Figures 3 e,f and S17) and is characterized by a more negative Δ*G*° (−49.3 kJ mol^−1^) than **A**. Accordingly, the assembly of **1** is more favorable than that of **A** and **B**, which might result from hydrogen bonding of the carboxylic acid groups in the hydrophobic interior of the aggregate. Furthermore, the fact that only the kinetically controlled aggregate **A** follows the isodesmic mechanism suggests that this pathway is mainly driven by aromatic and hydrophobic interactions, as typically observed for amphiphilic π‐systems that lack directional non‐covalent interaction patterns, such as hydrogen bonding.^17^, ^19^ On the contrary, such additional directional interactions are expected to stabilize the assemblies of **B** and **1** via a cooperative growth mechanism.^15^, ^20^

To unveil the relationship between assemblies **A** and **B**, time‐dependent UV/Vis experiments at different temperatures and concentrations were performed (Figures 3 g,h and S19). As also shown previously by UV/Vis, kinetically controlled species **A** readily converts to the thermodynamic product **B** simply by keeping the 20 μm aqueous aggregate solution of **A** at room temperature over the period of approximately one day (Figure 3 g). This transformation can be accelerated by increasing concentration and/or temperature (see plot of Abs_615_ vs. time in Figure 3 h), which is typical for the consecutive transformation of an on‐pathway species.^6^ Therefore, **A** converts into **B** via a rearrangement of the dye molecules within the aggregate, which does not involve the disassembly into free monomeric species. This transition between two precise aggregate species is also corroborated by a clear isosbestic point at 584 nm.

The resulting sigmoidal plots are diagnostic of an autocatalytic kinetic process,^21^ which is in accordance with the faster **A**→**B** transformation when **A** is allowed to coexist with small traces of **B** in solution (Figure S19). Also, the method used to isolate both aggregates starting from molecularly dissolved **2** differs significantly: aggregate **A** preferentially forms by rapid cooling (thermal quenching) or fast injection of the monomer solution into a large volume of water (solvophobic quenching). In contrast, the direct isolation of thermodynamic species **B** from the molecularly dissolved state is only possible by slow cooling (0.5 K min^−1^) of diluted monomer solutions (*c=*8 μm) using a 10 % volume fraction of THF in water. Unless a small percentage of THF is added, the aqueous assembly **B** will not dissociate into the monomer species, not even after heating the sample at a high temperature (368 K) for a prolonged time and using low concentrations (2.0 μm). The addition of THF lowers the energy barrier derived from the hydrophobic effect5j, 5l and therefore destabilizes the kinetically controlled species **A**, which is dictated by the hydrophobic collapse. Consequently, a direct transition from the monomer species to thermodynamic assembly **B** is facilitated via a new pathway, which does not involve the formation of kinetically controlled state **A**.

**Figure 4:**
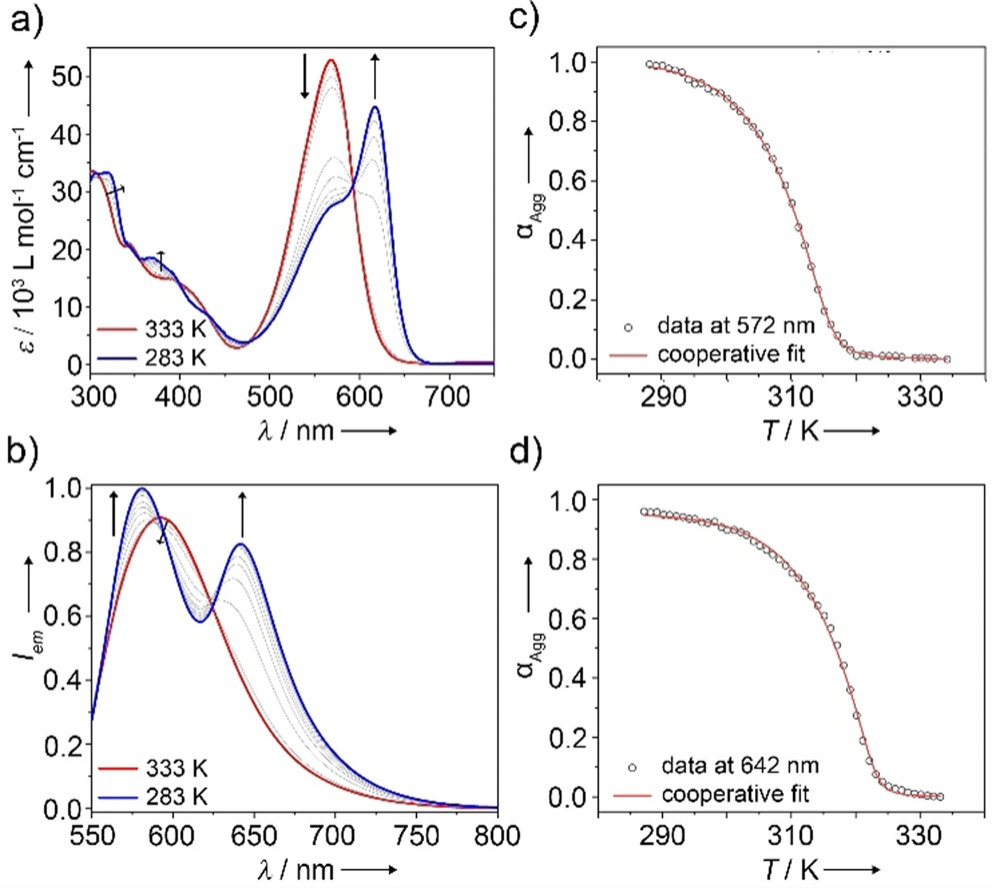
a) VT UV/Vis absorption and b) VT emission spectra of **B** (20 μm, water/THF (9:1) between 333 K and 283 K). *λ*_exc_=530 nm was used for emission studies. *α*_agg_ vs. *T* extracted from c) VT UV/Vis at *λ*=572 nm or d) from VT emission studies at 642 nm along with corresponding fits to the nucleation–elongation model.

After finding the appropriate experimental conditions [water/THF (9:1), 8 μm, cooling rate: 0.5 K min^−1^], the monomer‐to‐**B** transformation was monitored by variable‐temperature (VT) absorption and emission spectroscopy upon cooling monomer solutions from 333 K to 283 K. VT UV/Vis studies show the depletion of the monomer band at about 572 nm at the expense of the previously described J‐type aggregate band at 615 nm (Figure 4 a), which is the exact reverse behavior observed in denaturation experiments (Figure 3 e). The plot of *α*_agg_ vs. temperature extracted from these experiments was fitted to the cooperative nucleation–elongation model,^22^ yielding a Δ*G*° of −31.7 kJ mol^−1^ (Table S3, Figure 4 c). The VT emission spectra are in line with the absorption studies. Upon cooling, the single emission band located at about 590 nm decreases in intensity at the expense of two new aggregate bands at 580 nm and 641 nm (Figure 4 b). The changes in emission at 642 nm as a function of temperature were also satisfactorily fitted to the nucleation–elongation model, yielding thermodynamic parameters that are in good agreement with those obtained from VT absorption studies (Figure 4 c, Table S3).

The Δ*G*° values for **B** derived from VT spectroscopic studies are less negative than those extracted from previous denaturation experiments, which is not surprising considering the different experimental conditions used in both experiments: while the starting point for denaturation studies is the aggregate species in pure water, VT studies required the use of 10 % volume fraction of THF in order to disassemble the aggregates at high temperature, which obviously attenuates the overall aggregation tendency of the system. Furthermore, this energy difference can be related to the decrease of entropic contributions with the addition of a co‐solvent, as also recently reported by Würthner and co‐workers.19b

**Figure 5:**
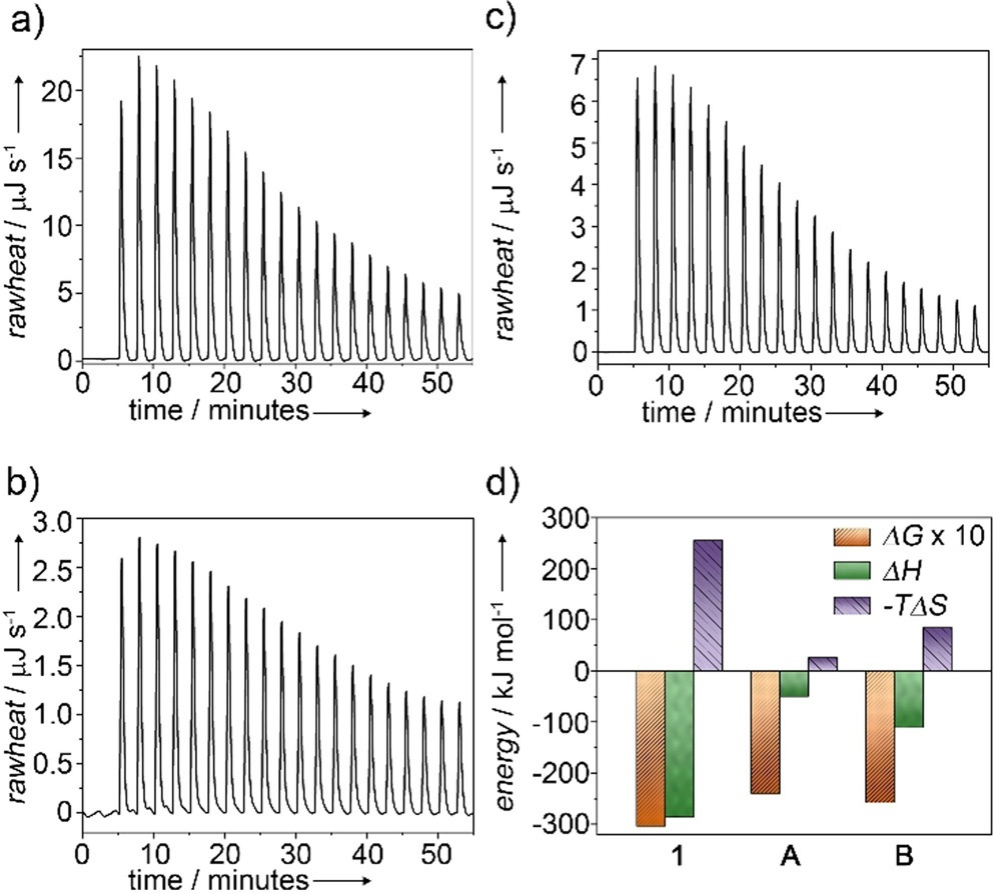
ITC dilution experiments of a) **1**, b) **A**, and c) **B**: injection of 1 mm water/THF (9:1) solution into pure water/THF (9:1) at 298 K and detected heat release. d) Comparison of entropic and enthalpic contributions to the overall Gibbs energy of **1**, **A**, and **B**.

To unravel the enthalpic and entropic contributions to the aggregation processes of the three species **1**, **A**, and **B**, isothermal titration calorimetry (ITC) dilution studies were recorded. Higher concentrations (1 mm) and small percentages of THF (10 %) had to be used in order to obtain sufficiently clear heat signals. The experiments were performed by addition of aliquots (2.5 μL) of the respective aggregate solutions to a larger volume (300 μL) of the pure solvent mixture [water/THF (9:1)]. For all three samples, dilution of the aggregate solution by injection into the solvent mixture water/THF (9:1) produced an endothermic heat flow due to the partial dissociation of the self‐assembled structure (Figure 5).^23^ The area of these raw heat signals was plotted against the injection number and fitted to the independent binding site model to determine the thermodynamic parameters of the respective disassembly process (Table S4, Figure S20). Under the investigated experimental conditions, all aggregates (**1**, **A**, **B**) are entropically disfavored and enthalpically favored (Figure 5 d). The entropic penalty related to these systems is expressed by the ratio between *T*Δ*S*° and Δ*H* ^298^ (Table S4). Among all aggregates, **A** exhibits the smallest entropic penalty (0.5) and the highest Gibbs energy (Δ*G* ^298^=−24.1 kJ mol^−1^), which highlights the key contribution of hydrophobic interactions to the self‐assembly process of **A**. This penalty increases after the transformation to species **B** is complete (0.8) and the enthalpy (Δ*H* ^298^) is lowered from −50.1 to −110.7 kJ mol^−1^, which results in an overall lower Gibbs energy (Δ*G* ^298^=−25.7 kJ mol^−1^). These much lower enthalpy values for **B** compared to **A** support our previous hypothesis that the assembly of **B** is stabilized by additional interaction patterns compared to **A**.

On the contrary, the assemblies of **1** are the energetically most favorable ones, as evident from their more negative Δ*G* ^298^ values (−30.3 kJ mol^−1^) compared to **A** and **B**. Furthermore, **1** exhibits the lowest Δ*H* ^298^ value by far (−286.1 kJ mol^−1^), which is in agreement with the existence of strong directional interactions, that is, pairwise interactions of carboxylic acids in the hydrophobic interior of the nanostructure. Also, the highest entropic penalty (0.9) obtained for this system reflects the high degree of order and rigidity of self‐assembled **1**. The thermodynamic parameters obtained by ITC differ from those extracted from previous denaturation studies (for instance, less negative Δ*G*° values for the former), as also observed when comparing denaturation and VT UV/Vis studies. This discrepancy can be explained by the use of 10 % THF and much higher concentrations (about two orders of magnitude) for ITC compared to denaturation studies. In contrast, comparable thermodynamic data are obtained by ITC and VT UV/Vis, as these experiments were performed in the same solvent mixture (10 % THF in water). However, for our particular system, we believe that VT UV/Vis is a more appropriate method to extract the thermodynamic parameters, as ITC is only able to cover a very small portion of the disassembly curve due to the much higher concentrations required for this measurement.

To rationalize the molecular packing of the assemblies, four dimers of **2** were optimized using the dispersion‐corrected semiempirical PM6 method in the vacuum (Figure S21, Table S5). In the most stable dimer structure, the BODIPY units are stacked in a slipped or J‐type fashion (*Θ*=26°, Figure S21) with a parallel orientation of the transition dipole moments^12^ (Scheme 2).^24^ The energy penalty associated with this parallel dye arrangement can be largely overcome by the formation of hydrogen bonds between the carboxylic acids along with strong π–π interactions between offset‐stacked BODIPY units. When this calculation is extended to a tetramer, it becomes obvious that a continuous hydrogen bonding network is not possible due to increased distances between the second and third molecule (3.8 Å, Figure S22). Thus, the extended π‐stacks with J‐type exciton coupling experimentally observed for **B** are most likely further stabilized by partial intermolecular hydrogen bonding between the carboxylic acid groups, as predicted by the calculations (see Scheme 2). Considering that **B** is not formed directly from the monomer in pure water but rather upon molecular rearrangement of the kinetically controlled species **A** (see Figure 3 g,h), the molecular packing of both aggregates should not differ significantly. Otherwise, disassembly of **A** into the monomeric species would be unavoidable prior to the formation of **B**. On this basis, a parallel arrangement of the BODIPY transition dipole moments can also be expected for **A**. Nevertheless, the lack of significant shifts in the absorption spectrum during the formation of **A** suggests weaker aromatic interactions than for **B**. Most likely, steric repulsion between the bulky benzaldehyde groups at the BODIPY *meso*‐position during the face‐to‐face approach of the monomer units prevents strong exciton coupling, increases π–π distances and ultimately hinders hydrogen bonding of the carboxylic acids (Scheme 2). This hypothesis is in line with the formation of disordered aggregates through an isodesmic mechanism (Figure 3 d), suggesting the absence of cooperative non‐covalent interactions, that is, hydrogen bonding.

On the contrary, for derivative **1**, the placement of the carboxyl group directly opposite of the TEG chains enables simultaneous pairwise interactions of the carboxylic groups via strong hydrogen bonding and 1D face‐to‐face stacking in the hydrophobic interior of the resulting nanostructure (see semiempirical calculations in Scheme 2 and Figure S23). Therefore, the lack of competition between entropic and enthalpic contributions in this system prevents pathway complexity.

**Figure 6:**
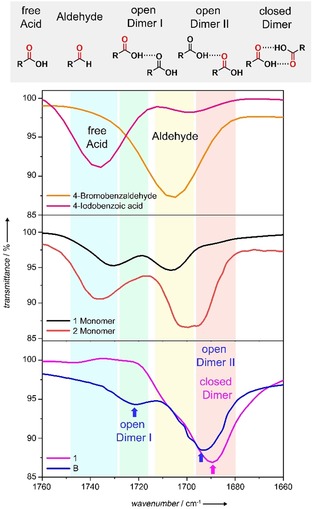
FTIR spectra of 4‐bromobenzaldehyde, 4‐iodobenzoic acid, a **1** monomer, a **2** monomer in DCM (0.5 mm), and **1** and **B** in thin films obtained from aqueous solutions at 298 K.

To substantiate our packing model, the existence of hydrogen bonding in both **1** and **B** was probed by Fourier‐transform infrared spectroscopy (FTIR) comparing thin films of the aggregate solutions in water and their respective monomer solutions in DCM. Prior to these measurements, we demonstrated by UV/Vis studies that the corresponding thermodynamic aggregates of **1** and **B** are retained upon thin‐film formation (Figure S24). Additionally, reference compounds (4‐bromobenzaldehyde and 4‐iodobenzoic acid) were also measured in DCM to identify the position of the most relevant bands (Figure 6). Compounds **1** and **2** in DCM (Figure 6, middle panel) exhibit characteristic carbonyl stretching bands of conjugated aldehydes ($\tilde{\nu }$ ≈ 1710 cm^−1^) and carboxylic acids ($\tilde{\nu }$ ≈1735 cm^−1^) that correspond to free, non‐hydrogen‐bonded groups. The position of these bands coincides approximately with that of the reference compounds (upper panel in Figure 6), indicating the existence of non‐hydrogen‐bonded carbonyl groups in all investigated molecules. Only for **2** in DCM, an additional band is observed at ≈1687 cm^−1^ (Figure 6, middle panel), which can be explained by the presence of hydrogen‐bonded carboxylic groups resulting from the high concentrations (0.5 mm) required for the FTIR measurement.

In contrast, for thin films of aggregate **1**, the carbonyl stretch of the free, non‐H‐bonded carboxylic acid groups disappears and a single band at $\tilde{\nu }$ =1689 cm^−1^ emerges (Figure 6, bottom panel), which is a distinctive feature of hydrogen‐bonded closed dimers of carboxylic acids.^25^ This behavior changes considerably for aggregate **B**, where the carbonyl stretch of the carboxyl groups splits into two bands at $\tilde{\nu }$ =1721 cm^−1^ and 1694 cm^−1^ (open dimer I and II, respectively; Figure 6, bottom panel). These results can be explained by an open acid‐dimer interaction pattern in which only one of the carbonyl groups of the dimer acts as a hydrogen‐bond acceptor in an interaction with the carboxyl OH of the second molecule, as depicted in Figure 6, top. The carbonyl group of the second molecule is consequently not involved in this interaction and therefore shifted to higher wavenumbers (open dimer I, $\tilde{\nu }$ ≈1721 cm^−1^). The slight shift of this band compared with the carbonyl stretching frequency of the free acid ($\tilde{\nu }$ ≈1735 cm^−1^) can be explained by a small electron‐withdrawing effect on the C=O group due to the involvement of the carboxyl OH as a hydrogen‐bond donor. Such an open acid‐dimer interaction has been previously identified for other systems in literature,^26^ showing identical patterns as in the present aggregates of **B**. These results help to rationalize the flexible nature of the fibers of **B** compared to the much more rigid nanostructures formed by **1**, since a closed dimer leads to drastic restrictions in the degrees of freedom, while an open dimer is highly dynamic.^27^

These findings support the previous hypothesis of an efficient hydrogen bonding arrangement for **1** that cannot be fully maintained for **B** without creating some defects, in agreement with the appearance of two energetic states in the packing of **B** in emission studies (Figure S12). The more favorable dimerization of **1** initially drives the self‐assembly into a more planar, better pre‐organized π‐system, where the hydrophobic aromatic groups can be efficiently shielded from the aqueous environment upon extended aggregation. In contrast, due to geometrical constraints, **2** is unable to form sufficiently stable closed hydrogen‐bonded carboxyl dimer seeds for extended supramolecular growth without exposing the hydrophobic content to the aqueous media. As a result, strong hydrophobic and aromatic interactions play a more prominent role than hydrogen bonding in the thermodynamic assembly of **B**. This is supported by the more significant broadening of the aromatic signals of **2** compared to **1** in ^1^H NMR studies when D_2_O is added to [D_8_]THF (Figure S25,S26). For both **1** and **B**, the aldehyde signal exhibits no deshielding, indicating the negligible role of this group in stabilizing both assemblies by hydrogen bonding, as also supported by FTIR (Figure S24).

## Conclusion

In conclusion, we have reported a new molecular design strategy for pathway control in aqueous self‐assembly that relies on the modification of the relative position of the substituents by keeping the molecular geometry of the building blocks unaltered. To this end, we have investigated the aqueous self‐assembly of two positional isomers of amphiphilic BODIPY dyes that solely differ in the position (2‐ or *meso*‐) of two functional groups (carboxyl and aldehyde). A number of experimental techniques (UV/Vis absorption and emission, FTIR, ITC, NMR, DLS, TEM, and cryo‐TEM) complemented by theoretical calculations reveal that the relative position of the carboxyl groups with respect to the glycol side chains (either about 90° or 180°) governs the existence of pathway complexity: typical pairwise interaction of the carboxyl groups occurs efficiently (in a local hydrophobic microenvironment) only if the carboxyl group is placed in‐line with the hydrophilic glycol side chains, resulting in a single thermodynamic cooperative lamellar assembly. In contrast, attachment of the carboxyl group at the *meso*‐position induces competition of entropic and enthalpic contributions, leading to a kinetically controlled isodesmic pathway that rearranges over time into thermodynamically controlled nanofibers. For this assembly, simultaneous J‐type exciton coupling of the BODIPY dyes and partial hydrogen bonding of the carboxyl groups induce a cooperative growth mechanism. Our results reveal that rational placement of functional groups for weak interactions is a prerequisite for pathway selection in aqueous self‐assembly. We believe that our design strategy could be extended to various types of molecular platforms where substituents can be arranged in a variety of defined angles.

## Supporting information

As a service to our authors and readers, this journal provides supporting information supplied by the authors. Such materials are peer reviewed and may be re‐organized for online delivery, but are not copy‐edited or typeset. Technical support issues arising from supporting information (other than missing files) should be addressed to the authors.

Supplementary

## References

[1] J. A. Marsh , S. A. Teichmann , Annu. Rev. Biochem. 2015, 84, 551. 25494300 10.1146/annurev-biochem-060614-034142

[2] D. L. D. Caspar , C. Cohen , W. Longley , J. Mol. Biol. 1969, 41, 87. 5803288 10.1016/0022-2836(69)90128-4

[3a] P. A. Korevaar , T. F. A. de Greef , E. W. Meijer , Chem. Mater. 2014, 26, 576;

[3b] P. A. Korevaar , S. J. George , A. J. Markvoort , M. M. J. Smulders , P. A. J. Hilbers , A. P. H. J. Schenning , T. F. A. de Greef , E. W. Meijer , Nature 2012, 481, 492. 22258506 10.1038/nature10720

[4a] A. Langenstroer , K. K. Kartha , Y. Dorca , J. Droste , V. Stepanenko , R. Q. Albuquerque , M. R. Hansen , L. Sánchez , G. Fernández , J. Am. Chem. Soc. 2019, 141, 5192; 30785744 10.1021/jacs.8b11011

[4b] M. Wehner , M. I. S. Röhr , M. Bühler , V. Stepanenko , W. Wagner , F. Würthner , J. Am. Chem. Soc. 2019, 141, 6092; 30892890 10.1021/jacs.9b02046

[4c] E. E. Greciano , B. Matarranz , L. Sánchez , Angew. Chem. Int. Ed. 2018, 57, 4697; 10.1002/anie.201801575 29474002

[4d] T. Fukui , S. Kawai , S. Fujinuma , Y. Matsushita , T. Yasuda , T. Sakurai , S. Seki , M. Takeuchi , K. Sugiyasu , Nat. Chem. 2017, 9, 493; 28430199 10.1038/nchem.2684

[4e] S. Ogi , V. Stepanenko , J. Thein , F. Würthner , J. Am. Chem. Soc. 2016, 138, 670; 26699283 10.1021/jacs.5b11674

[4f] S. Ogi , V. Stepanenko , K. Sugiyasu , M. Takeuchi , F. Würthner , J. Am. Chem. Soc. 2015, 137, 3300; 25689054 10.1021/ja511952c

[4g] F. Aparicio , B. Nieto-Ortega , F. Nájera , F. J. Ramírez , J. T. López Navarrete , J. Casado , L. Sánchez , Angew. Chem. Int. Ed. 2014, 53, 1373; 10.1002/anie.201309172 24352979

[4h] S. Ogi , T. Fukui , M. L. Jue , M. Takeuchi , K. Sugiyasu , Angew. Chem. Int. Ed. 2014, 53, 14363; 10.1002/anie.201407302 25354399

[5a] E. Cohen , H. Weissman , I. Pinkas , E. Shimoni , P. Rehak , P. Král , B. Rybtchinski , ACS Nano 2018, 12, 317; 29257866 10.1021/acsnano.7b06376

[5b] B. Kemper , L. Zengerling , D. Spitzer , R. Otter , T. Bauer , P. Besenius , J. Am. Chem. Soc. 2018, 140, 534; 29271649 10.1021/jacs.7b08189

[5c] R. P. M. Lafleur , X. Lou , G. M. Pavan , A. R. A. Palmans , E. W. Meijer , Chem. Sci. 2018, 9, 6199; 30090307 10.1039/c8sc02257g PMC6062890

[5d] N. M. Matsumoto , R. P. M. Lafleur , X. Lou , K.-C. Shih , S. P. W. Wijnands , C. Guibert , J. W. A. M. van Rosendaal , I. K. Voets , A. R. A. Palmans , Y. Lin , E. W. Meijer , J. Am. Chem. Soc. 2018, 140, 13308; 30221520 10.1021/jacs.8b07697 PMC6194755

[5e] S. Ogi , C. Grzeszkiewicz , F. Würthner , Chem. Sci. 2018, 9, 2768; 29732062 10.1039/c7sc03725b PMC5914135

[5f] A. Singh , J. P. Joseph , D. Gupta , I. Sarkar , A. Pal , Chem. Commun. 2018, 54, 10730; 10.1039/c8cc06266h 30191235

[5g] Z. Chen , Y. Liu , W. Wagner , V. Stepanenko , X. Ren , S. Ogi , F. Würthner , Angew. Chem. Int. Ed. 2017, 56, 5729; 10.1002/anie.201701788 28371081

[5h] A. Aliprandi , M. Mauro , L. de Cola , Nat. Chem. 2016, 8, 10; 26673259 10.1038/nchem.2383

[5i] F. Tantakitti , J. Boekhoven , X. Wang , R. V. Kazantsev , T. Yu , J. Li , E. Zhuang , R. Zandi , J. H. Ortony , C. J. Newcomb , L. C. Palmer , G. S. Shekhawat , M. O. de La Cruz , G. C. Schatz , S. I. Stupp , Nat. Mater. 2016, 15, 469; 26779883 10.1038/nmat4538 PMC4805452

[5j] J. Baram , H. Weissman , Y. Tidhar , I. Pinkas , B. Rybtchinski , Angew. Chem. Int. Ed. 2014, 53, 4123; 10.1002/anie.201310571 24644217

[5k] P. A. Korevaar , C. J. Newcomb , E. W. Meijer , S. I. Stupp , J. Am. Chem. Soc. 2014, 136, 8540; 24911245 10.1021/ja503882s

[5l] Y. Tidhar , H. Weissman , S. G. Wolf , A. Gulino , B. Rybtchinski , Chem. Eur. J. 2011, 17, 6068; 21542033 10.1002/chem.201003419

[5m] J.-K. Kim , E. Lee , M.-C. Kim , E. Sim , M. Lee , J. Am. Chem. Soc. 2009, 131, 17768; 19921778 10.1021/ja907462h

[5n] J. Bae , J.-H. Choi , Y.-S. Yoo , N.-K. Oh , B.-S. Kim , M. Lee , J. Am. Chem. Soc. 2005, 127, 9668. 15998054 10.1021/ja051961m

[6] J. Matern , Y. Dorca , L. Sánchez , G. Fernandez , Angew. Chem. Int. Ed. 2019, 58, 16730; 10.1002/anie.201905724 PMC6900041 31271244

[7a] T. S. Kale , H. A. M. Ardoña , A. Ertel , J. D. Tovar , Langmuir 2019, 35, 2270; 30642185 10.1021/acs.langmuir.8b03708

[7b] V. Saez Talens , D. M. M. Makurat , T. Liu , W. Dai , C. Guibert , W. E. M. Noteborn , I. K. Voets , R. E. Kieltyka , Polym. Chem. 2019, 10, 3146;

[7c] P. Dey , P. Rajdev , P. Pramanik , S. Ghosh , Macromolecules 2018, 51, 5182;

[7d] P. Pramanik , D. Ray , V. K. Aswal , S. Ghosh , Angew. Chem. Int. Ed. 2017, 56, 3516; 10.1002/anie.201611715 28211226

[7e] S. K. Albert , M. Golla , H. V. P. Thelu , N. Krishnan , P. Deepak , R. Varghese , Org. Biomol. Chem. 2016, 14, 6960; 27241196 10.1039/c6ob00681g

[7f] T. S. Kale , J. D. Tovar , Tetrahedron 2016, 72, 6084;

[7g] E. Krieg , M. M. C. Bastings , P. Besenius , B. Rybtchinski , Chem. Rev. 2016, 116, 2414; 26727633 10.1021/acs.chemrev.5b00369

[7h] R. Appel , J. Fuchs , S. M. Tyrrell , P. A. Korevaar , M. C. A. Stuart , I. K. Voets , M. Schönhoff , P. Besenius , Chem. Eur. J. 2015, 21, 19257; 26555139 10.1002/chem.201503616

[7i] V. Saez Talens , P. Englebienne , T. T. Trinh , W. E. M. Noteborn , I. K. Voets , R. E. Kieltyka , Angew. Chem. Int. Ed. 2015, 54, 10502; 10.1002/anie.201503905 26179942

[7j] S. K. Albert , H. V. P. Thelu , M. Golla , N. Krishnan , S. Chaudhary , R. Varghese , Angew. Chem. Int. Ed. 2014, 53, 8352; 10.1002/anie.201403455 24962762

[7k] G. L. Eakins , J. K. Gallaher , R. A. Keyzers , A. Falber , J. E. A. Webb , A. Laos , Y. Tidhar , H. Weissman , B. Rybtchinski , P. Thordarson , J. M. Hodgkiss , J. Phys. Chem. B 2014, 118, 8642; 24950450 10.1021/jp504564s

[7l] G. Fernández , F. García , F. Aparicio , E. Matesanz , L. Sánchez , Chem. Commun. 2009, 7155; 10.1039/b916418a 19921014

[7m] X. Zhang , Z. Chen , F. Würthner , J. Am. Chem. Soc. 2007, 129, 4886; 17402739 10.1021/ja070994u

[7n] B.-S. Kim , D.-J. Hong , J. Bae , M. Lee , J. Am. Chem. Soc. 2005, 127, 16333. 16287329 10.1021/ja055999a

[8] A. Loudet , K. Burgess , Chem. Rev. 2007, 107, 4891. 17924696 10.1021/cr078381n

[9a] D. S. Pal , H. Kar , S. Ghosh , Chem. Commun. 2018, 54, 928; 10.1039/c7cc08302e 29318226

[9b] M. R. Molla , D. Gehrig , L. Roy , V. Kamm , A. Paul , F. Laquai , S. Ghosh , Chem. Eur. J. 2014, 20, 760. 24339217 10.1002/chem.201303379

[10] M. R. Molla , S. Ghosh , Phys. Chem. Chem. Phys. 2014, 16, 26672. 25375094 10.1039/c4cp03791j

[11] S. Kim , J. Bouffard , Y. Kim , Chem. Eur. J. 2015, 21, 17459. 26463266 10.1002/chem.201503040

[12] A similar packing with unfavorable parallel dipole moment and slipped arrangement of the BODIPY groups was previously observed. The electron-withdrawing CF_3_ substituent at the *meso*-position stabilizes this parallel dye packing: S. Choi , J. Bouffard , Y. Kim , Chem. Sci. 2014, 5, 751.

[13] F. Würthner , T. E. Kaiser , C. R. Saha-Möller , Angew. Chem. Int. Ed. 2011, 50, 3376; 10.1002/anie.201002307 21442690

[14] A. Rödle , B. Ritschel , C. Mück-Lichtenfeld , V. Stepanenko , G. Fernández , Chem. Eur. J. 2016, 22, 15772. 27650885 10.1002/chem.201602592

[15] A. Rödle , M. Lambov , C. Mück-Lichtenfeld , V. Stepanenko , G. Fernández , Polymer 2017, 128, 317.

[16] C. Rest , A. Martin , V. Stepanenko , N. K. Allampally , D. Schmidt , G. Fernández , Chem. Commun. 2014, 50, 13366. 10.1039/c4cc06152g 25232799

[17] F. García , G. Fernández , L. Sánchez , Chem. Eur. J. 2009, 15, 6740. 19499561 10.1002/chem.200900303

[18] P. A. Korevaar , C. Schaefer , T. F. A. de Greef , E. W. Meijer , J. Am. Chem. Soc. 2012, 134, 13482. 22808949 10.1021/ja305512g

[19a] P. P. N. Syamala , B. Soberats , D. Görl , S. Gekle , F. Würthner , Chem. Sci. 2019, 10, 884; 32110300 10.1039/c9sc03103k PMC7017873

[19b] D. Görl , F. Würthner , Angew. Chem. Int. Ed. 2016, 55, 12094; 10.1002/anie.201606917 27558471

[19c] N. K. Allampally , A. Florian , M. J. Mayoral , C. Rest , V. Stepanenko , G. Fernández , Chem. Eur. J. 2014, 20, 10669; 25042858 10.1002/chem.201402077

[19d] M. J. Mayoral , C. Rest , J. Schellheimer , V. Stepanenko , G. Fernández , Chem. Eur. J. 2012, 18, 15607; 23132726 10.1002/chem.201202367

[19e] F. García , L. Sánchez , Chem. Eur. J. 2010, 16, 3138. 20119992 10.1002/chem.200902894

[20a] S. Ogi , N. Fukaya , Arifin , B. B. Skjelstad , Y. Hijikata , S. Yamaguchi , Chem. Eur. J. 2019, 25, 7303; 30916444 10.1002/chem.201901382

[20b] A. Sampedro , Á. Ramos-Torres , C. Schwöppe , C. Mück-Lichtenfeld , I. Helmers , A. Bort , I. Díaz-Laviada , G. Fernández , Angew. Chem. Int. Ed. 2018, 57, 17235; 10.1002/anie.201804783 30324638

[20c] C. Rest , R. Kandanelli , G. Fernández , Chem. Soc. Rev. 2015, 44, 2543; 25735967 10.1039/c4cs00497c

[20d] F. García , J. Buendía , L. Sánchez , J. Org. Chem. 2011, 76, 6271. 21692521 10.1021/jo201055t

[21] P. A. Bachmann , P. L. Luisi , J. Lang , Nature 1992, 357, 57.

[22a] H. M. M. ten Eikelder , A. J. Markvoort , T. F. A. de Greef , P. A. J. Hilbers , J. Phys. Chem. B 2012, 116, 5291; 22443806 10.1021/jp300622m

[22b] A. J. Markvoort , H. M. M. ten Eikelder , P. A. J. Hilbers , T. F. A. de Greef , E. W. Meijer , Nat. Commun. 2011, 2, 509. 22027589 10.1038/ncomms1517 PMC3207207

[23] A. Arnaud , L. Bouteiller , Langmuir 2004, 20, 6858. 15274596 10.1021/la049365d

[24] F. C. Spano , Acc. Chem. Res. 2010, 43, 429. 20014774 10.1021/ar900233v

[25a] B. Matarranz , A. Sampedro , C. G. Daniliuc , G. Fernández , Crystals 2018, 8, 436;

[25b] M. Takasuka , K. Matsumura , N. Ishizuka , Vib. Spectrosc. 2001, 25, 63.

[26a] M. J. Abdy , A. Murdoch , A. Martínez-Felipe , Liq. Cryst. 2016, 43, 2191;

[26b] A. Martinez-Felipe , A. G. Cook , J. P. Abberley , R. Walker , J. M. D. Storey , C. T. Imrie , RSC Adv. 2016, 6, 108164;

[26c] A. Martínez-Felipe , A. G. Cook , M. J. Wallage , C. T. Imrie , Phase Transitions 2014, 87, 1191;

[26d] J. Chen , C. L. Brooks , H. A. Scheraga , J. Phys. Chem. B 2008, 112, 242; 17880128 10.1021/jp074355h PMC2561919

[26e] M. Petrov , E. Anachkova , N. Kirov , H. Ratajczak , J. Baran , J. Mol. Liq. 1994, 61, 221.

[27] A. Martínez-Felipe , J. D. Badia , L. Santonja-Blasco , C. T. Imrie , A. Ribes-Greus , Eur. Polym. J. 2013, 49, 1553.

