# Supplementary material for: Impact of Positional Isomerism on Pathway Complexity in Aqueous Media
Source: Angew Chem Int Ed Engl. 2020 Feb 3;59(14):5675–82. doi: 10.1002/anie.201911531 (PMC7154731; doi:10.1002/anie.201911531)
Supplement: Supplementary file 1 — Supplementary [file ANIE-59-5675-s001.pdf]

Angewandte Chemie
Eine Zeitschrift der Gesellschaft Deutscher Chemiker

Supporting Information

Impact of Positional Isomerism on Pathway Complexity in Aqueous Media

*Ingo Helmers, Bowen Shen, Kalathil K. Kartha, Rodrigo Q. Albuquerque, Myongsoo Lee, and Gustavo Fernández**

anie_201911531_sm_miscellaneous_information.pdf

Supporting Information

Table of Contents

Experimental Procedure **S3**

Materials and Methods **S3**

Synthetic details and characterization **S4-S13**

Results and Discussion **S14-S30**

Nucleation-Elongation model **S14**

Denaturation model ***S14***

Fluorescence Quantum Yield ***S15***

Thermodynamic Parameters **S16**

Quantum Chemical Calculations ***S17***

Supplementary Figures ***S17-S29***

References ***S29***

Experimental Procedure

Materials and Methods

Chemical and Reagents: All chemicals were purchased from Sigma-Aldrich (St. Louis, MO, USA), TCI Europe N.V. (Tokyo, JP) or Alfa Aesar (Ward Hill, MA, USA), with minimum analytical grade quality and used without further purification unless otherwise stated. Dichloromethane was pre-dried over $CaCl_2$ and then distilled over P_2O_5 under argon atmosphere. Silica gel was used for column chromatography unless otherwise indicated.

Column chromatography. Preparative column chromatography was conducted in self-packed glass columns of different sizes with silica gel (particle size: 40 – 60 µm, *Merck*). Dichloromethane and methanol were distilled before usage.

NMR spectroscopy: ^{1}H and ^{13}C NMR spectra were recorded at 298 K on Avance II 300 and Avance II 400 from Bruker for routine experiments using tetramethylsilane (TMS) as internal reference, and DD2 500 and DD2 600 from Agilent for characterization purposes. Multiplicities for proton signals are abbreviated as s, d, t, q and m for singlet, doublet, triplet, quadruplet and multiplet, respectively.

Mass spectrometry: ESI mass spectra were measured on a Bruker MicrOToF system.

UV/Vis and fluorescence spectroscopy: UV/Vis absorption spectra were recorded on a Jasco V-770 or a Jasco V-750 spectrophotometers, both equipped with peltier cells and Julabo F250 water circulation units. Fluorescence spectra were recorded on a Jasco FP-8500 spectrofluorimeter equipped with the same water circulation unit.

FT-IR spectroscopy: Solution and solid-state measurements were carried out using a JASCO-FT-IR-6800 and a CaF_2 cell with a path length of 0.1 mm. For all measurements, solvents of spectroscopic grade (UVasol) from Merck were used.

Dynamic Light Scattering: DLS measurements were performed on a CGS-3 Compact Goniometer System from ALV, equipped with a LSE-5004 Light Scattering Electronics (22 mW HeNe Laser (633 nm)) and Multiple Tau Digital Correlator unit from ALV. Solvents were filtered prior to sample preparation through nylon or Teflon filters with a pore size of 0.45 µm.

TEM experiments: To investigate the self-assembled structures in aqueous solution, a drop of each sample solution was placed on a carbon-coated copper grid (Carbon Type B (15-25 nm) on 200 mesh, with Formvar; Ted Pella, Inc.) and the solution was allowed to evaporate under ambient conditions. These samples were stained by depositing a drop of uranyl acetate aqueous solution (0.4-1.0 wt %) onto the surface of the sample-loaded grid. The dried specimen was observed by a JEOL-JEM HR2100 operated at 120 kV with a Dual vision 300 W and SC 1000 CCD camera (Gatan, Inc.; Warrendale, PA). The data were analyzed using Digital Micrograph software. The cryogenic transmission electron microscopy (cryo-TEM) experiments were performed with a thin film of aqueous solution of amphiphiles (3 µl) transferred to a lacey supported grid. The thin aqueous films were prepared under controlled temperature (25 °C) and humidity conditions (97-99 %) within a custom-built environmental chamber in order to prevent evaporation of water from sample solution (FEI Vitrobot Mark IV). The excess liquid was blotted with filter paper for 2-3 seconds, and the thin aqueous films were rapidly vitrified by plunging them into liquid ethane (cooled by liquid nitrogen) at its freezing point. The grid was transferred on a Gatan 626 cryo holder, using a cryo-transfer device and transferred to the FEI Talos-F200C with a Ceta 16M CMOS camera. Direct imaging was carried out at a temperature of approximately -175 °C and with a 200 kV accelerating voltage. The data were analyzed using TEM Imaging & Analysis software.

Sample preparation method: Aggregates of **1** and **2** were dissolved in water under sonication (nominal power: 80 W, frequency: 35 kHz) at room temperature for 5 minutes. Whenever THF was used as co-solvent, samples in pure water were diluted with the corresponding volume fraction of THF to obtain the desired final concentration.

Dilution ITC experiments: A TA Instruments Nano ITC Low Volume (Waters Corp., Milford, Massachusetts, USA) with ITCRun Version 2.1.7.0 Firmware version 1.31 (TA Instruments, Waters Corp., Milford, Massachusetts, USA) as software was used for ITC dilution experiments. A solution of the respective sample at 1 mM in water/THF (9/1) was titrated using a 50 µL syringe in 20 injections of 2.5 µL at a temperature of 25 °C and a stirring rate of 350 rpm into 300 µL of a solvent mixture water/THF (9/1). The data were fitted using the independent model of NanoAnalyse Data Analysis version 2.36 (TA Instruments, Waters Corp., Milford, Massachusetts, USA). Solutions of **A** were measured immediately after preparation, since the transformation rate to **B** increases with higher concentrations.

Synthesis and characterization of 1 and 2

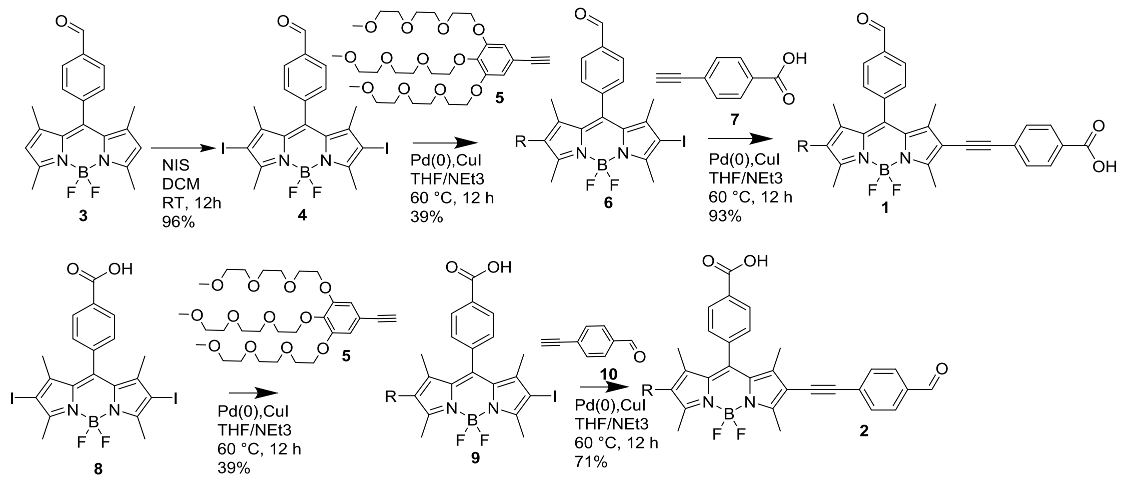

4-(5,5-difluoro-2,8-diiodo-1,3,7,9-tetramethyl-5*H*-4λ^4,5λ^4-dipyrrolo[1,2-c:2',1'-f][1,3,2]diazaborinin-10-yl)benzoic acid (**8**),[1] 4-(5,5-difluoro-1,3,7,9-tetramethyl-5*H*-4λ^4,5λ^4-dipyrrolo[1,2-c:2',1'-f][1,3,2]diazaborinin-10-yl)benzaldehyde (**3**),[2] 4-ethynylbenzoic acid (**7**),[3] 4-ethynylbenzaldehyde (**10**)[4] and 5-ethynyl-1,2,3-tris(2-(2-(2-methoxyethoxy)ethoxy)ethoxy)-benzene (**5**)[5] were prepared following reported synthetic procedures and showed identical spectroscopic properties to those reported therein.

Synthesis of 4-(5,5-difluoro-2-iodo-1,3,7,9-tetramethyl-8-((3,4,5-tris(2-(2-(2-methoxyethoxy)ethoxy)ethoxy)phenyl)-ethynyl)5*H*-5λ^4,6λ^4-dipyrrolo[1,2-c:2',1'-f][1,3,2]diazaborinin-10-yl)benzoic acid (9)

4-(5,5-difluoro-2,8-diiodo-1,3,7,9-tetramethyl-5*H*-4λ^4,5λ^4-dipyrrolo[1,2-c:2',1'-f][1,3,2]diazaborinin-10-yl)benzoic acid (**8**, 1.95 g, 3.16 mmol, 1 eq), $(PPh_3)_4Pd$ (0.18 g, 0.16 mmol, 0.05 eq) and CuI (30.1 mg, 0.16 mmol, 0.05 eq) were dissolved in a mixture of anhydrous THF (10 mL) and NEt_3 (10 mL) and subjected to three vacuum-argon cycles The reaction mixture was heated to 50 °C and 5-ethynyl-1,2,3-tris(2-(2-(2-methoxyethoxy)ethoxy)ethoxy)benzene (**5,** 1.86 g, 3.16 mmol, 1.0 eq) dissolved in THF (100 mL) was added dropwise over the course of 2 hours to the solution and stirred for additional 12 h at 50°C. All solvents were removed and the crude product was purified by silica gel column chromatography using a gradient from dichloromethane (DCM) to DCM/MeOH (8/2, (*v*/*v*)) as eluent, followed by reverse phase (C18) column chromatography using a gradient from water/ACN (9/1, (*v*/*v*)) to ACN as eluent.
Yield: 1.30 g (**9**, 1.22 mmol, 39%) of a black solid.

<u>Characterization of **9**</u>:

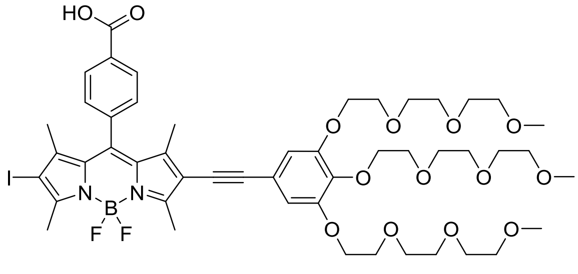

Chemical Formula: $C_{49}H_{64}BF_2IN_2O_{13}$
Exact Mass: 1064.3514
Molecular Weight: 1064.7633

^{1}H NMR (400 MHz, $CDCl_3$): δ (in ppm) = 8.20 (m, 2H-Ar), 7.30 (m, 2H-Ar), 6.66 (s, 2H-Ar), 4.30 – 4.03 (m, 6H-TEG), 3.98 – 3.46 (m, 30H-TEG), 3.35 (m, 9H-TEG), 2.80 – 2.54 (m, 6H-BODIPY), 1.64 – 1.29 (m, 6H-BODIPY).
^{13}C NMR (101 MHz, $CDCl_3$): δ (in ppm) = 158.9, 158.8, 156.9, 153.9, 152.4, 144.8, 144.1, 141.6, 141.2, 140.1, 138.9, 137.9, 131.6, 130.9, 130.5, 130.3, 127.7, 118.4, 116.2, 111.9, 110.9, 96.4, 85.7, 80.6, 72.3, 71.9, 71.8, 70.7, 70.6, 70.5, 70.4, 70.3, 69.5, 68.8, 59.0, 59.0, 53.5, 29.7, 17.0, 16.0, 13.8, 13.7.
ESI-MS (TOF)**:** *m/z* 1103.3356 $[M+Na]^+$, calculated for $C_{49}H_{64}N_2O_{14}BF_2INa$: 1103.33628

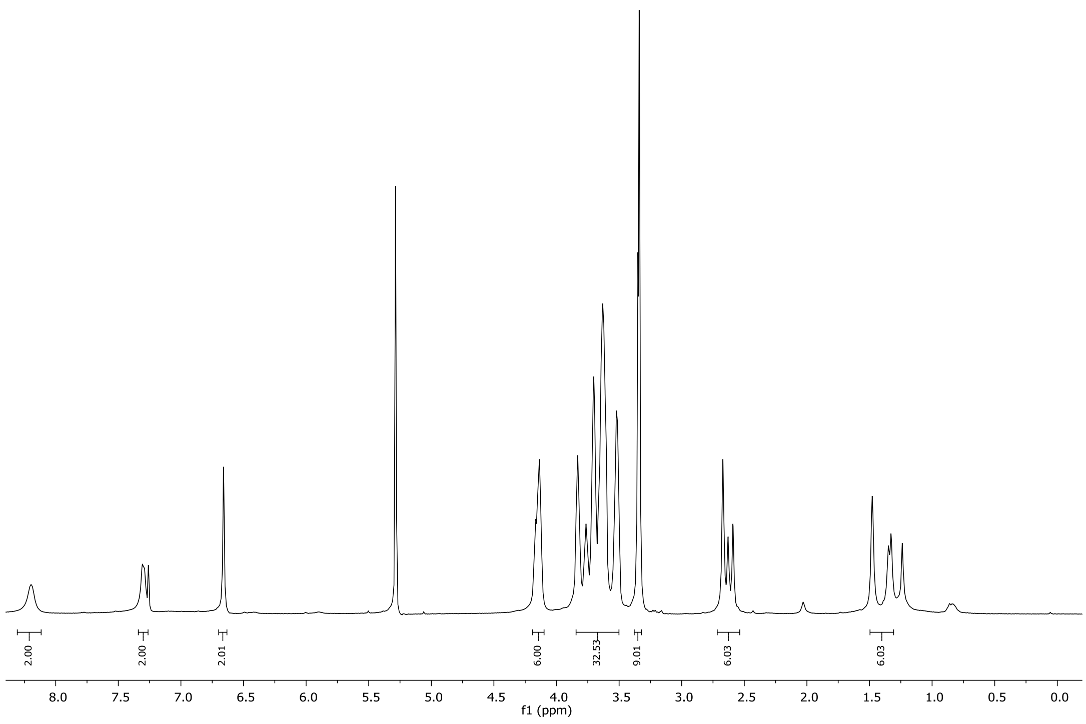

Figure S1. ^{1}H NMR (400 MHz, $CDCl_3$, 298 K) of **9**.

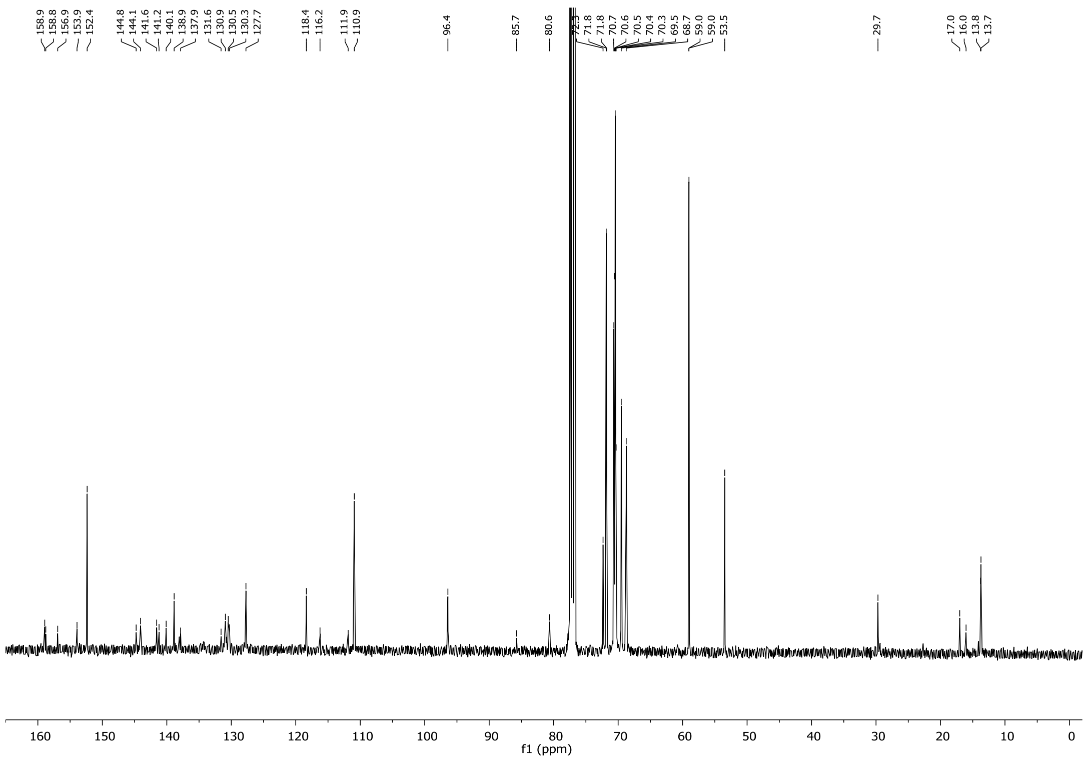

Figure S2. ^{13}C NMR (101 MHz, $CDCl_3$, 298 K) of compound **9**.

4-(5,5-difluoro-2-((4-formylphenyl)ethynyl)-1,3,7,9-tetramethyl-8-((3,4,5-tris(2-(2-(2-methoxyethoxy)ethoxy)ethoxy)phenyl)ethynyl)-5*H*-5λ^4,6λ^4-dipyrrolo[1,2-c:2',1'-f][1,3,2]diazaborinin-10-yl)benzoic acid (2)

4-(5,5-difluoro-2-iodo-1,3,7,9-tetramethyl-8-((3,4,5-tris(2-(2-(2-methoxyethoxy)ethoxy)ethoxy)phenyl)ethynyl)-5*H*-5λ^4,6λ^4-dipyrrolo[1,2-c:2',1'-f][1,3,2]diazaborinin-10-yl)benzoic acid (**9**, 531 mg, 0.49 mmol, 1 eq), $(PPh_3)_4Pd$ (28.4 mg, 24.6 µmol, 0.05 eq) and CuI (4.6 mg, 24.6 µmol, 0.05 eq) were dissolved in a mixture of anhydrous THF (20 mL) and NEt_3 (10 mL) and subjected to three vacuum-argon cycles The reaction mixture was then heated to 50 °C and 4-ethynylbenzaldehyde (**10,** 96.0 mg, 0.74 mmol, 1.5 eq) was added to the solution and stirred for additional 12 h at 50°C. The solvent was removed and the crude product purified by silica gel column chromatography using a gradient from DCM to DCM/MeOH (20 %) as eluent, followed by reverse phase (C18) column chromatography using a gradient from water/ACN (9/1, (*v*/*v*)) to ACN as eluent.
Yield = 378 mg (**2**, 0.35 mmol, 72%) of a blue solid.

Characterization of **2**:

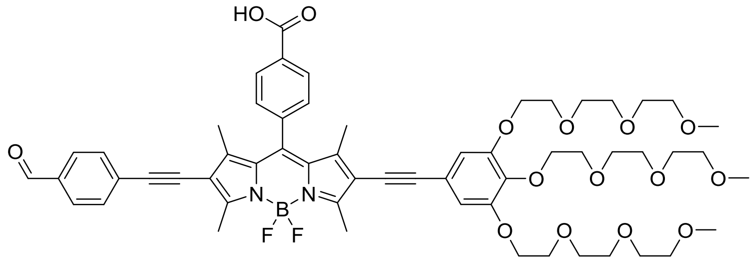

Chemical Formula: $C_{58}H_{69}BF_2N_2O_{15}$
Exact Mass: 1082.4759
Molecular Weight: 1082.9958

^{1}H NMR (500 MHz, $CDCl_3$): δ (in ppm) = 9.97 (s, 1H-ALD), 8.26 (d, *J* = 7.8 Hz, 2H-Ar), 7.81 (d, *J* = 8.4 Hz, 2H-Ar), 7.56 (d, *J* = 8.3 Hz, 2H-Ar), 7.42 (d, *J* = 8.0 Hz, 2H-Ar), 6.68 (s, 2H-Ar-TEG), 4.23 – 4.11 (m, 6H-TEG), 3.96 – 3.60 (m, 24H-TEG), 3.57 – 3.47 (m, 6H-TEG), 3.35 (m, 9H-TEG), 2.71 (m, 6H-BODIPY), 1.48 (m, 6H-BODIPY).
^{13}C NMR (126 MHz, $CDCl_3$): δ (in ppm) = 191.5, 169.3, 159.9, 158.6, 152.6, 152.6, 144.2, 143.8, 141.3, 139.5, 139.4, 135.3, 132.3, 131.7, 131.5, 131.2, 130.7, 129.7, 128.6, 128.4, 118.0, 117.1, 115.6, 111.2, 97.1, 96.1, 86.1, 80.3, 59.1, 53.6, 53.4, 53.1, 52.9, 52.7, 29.8, 14.0, 13.8, 13.8, 13.6.
ESI-MS (TOF)**:** *m*/*z* 1105.46609 $[M+Na]^+$, calculated for $C_{58}H_{69}N_2O_{15}BF_2$ Na: 1105.46644

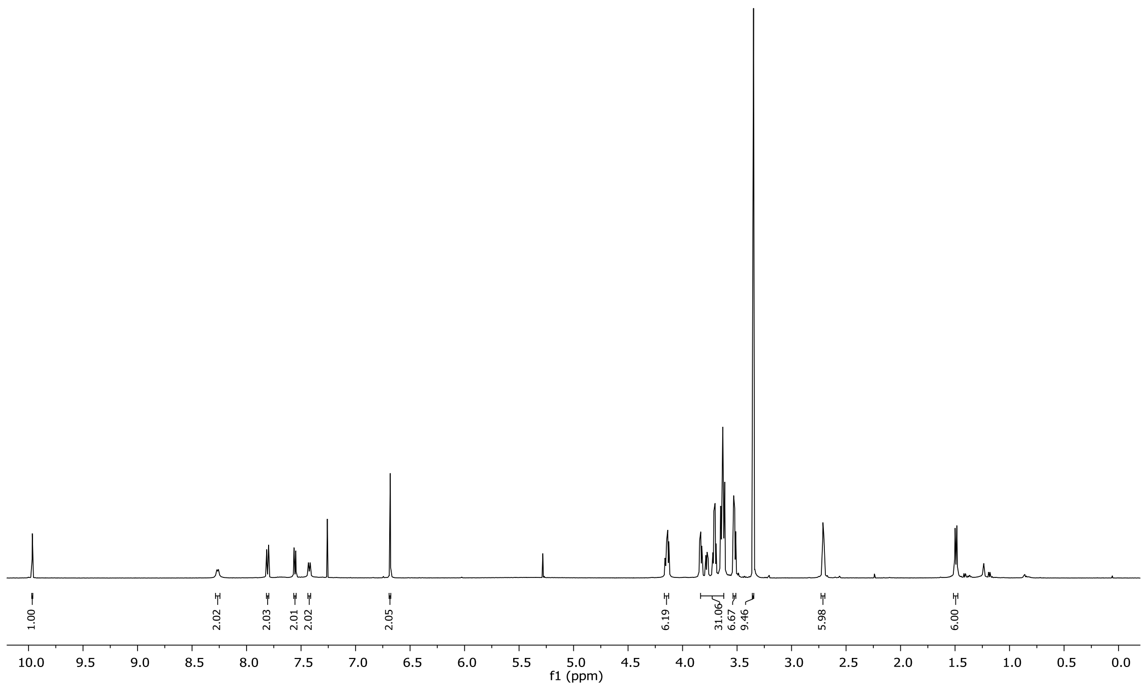

Figure S3. ^{1}H NMR (500 MHz, $CDCl_3$, 298 K) of **2**.

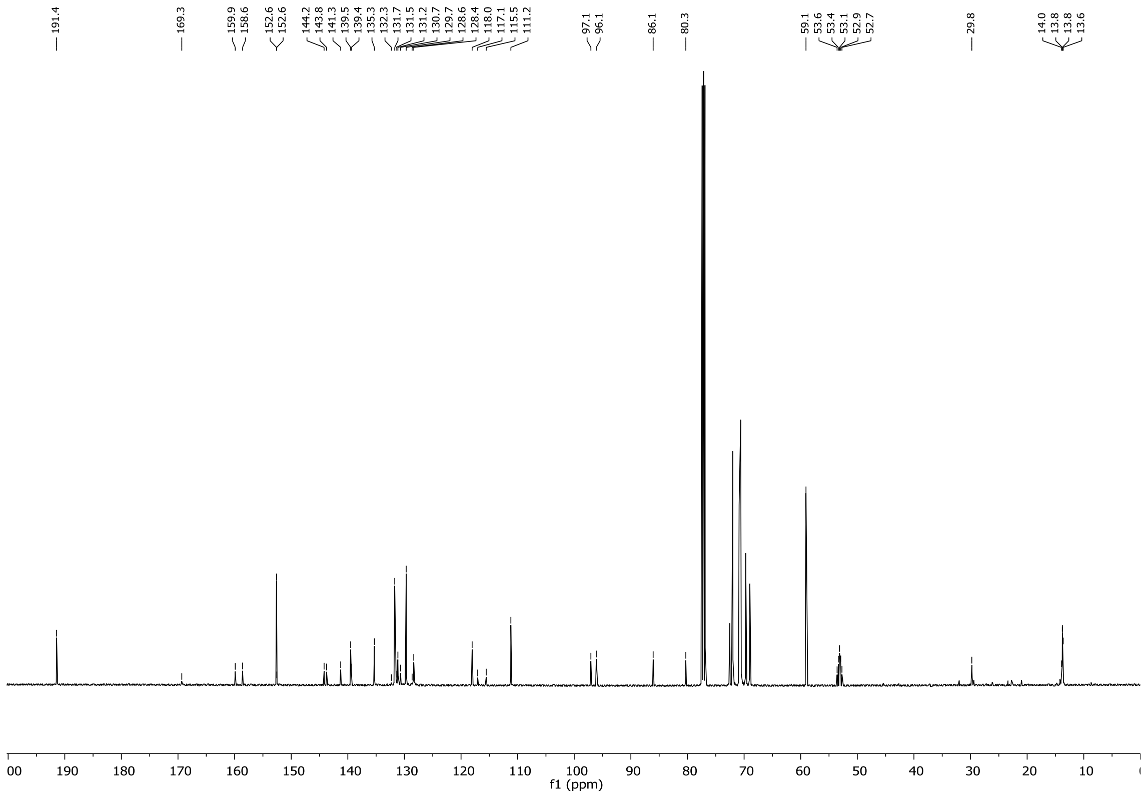

Figure S4. ^{13}C NMR (125 MHz, $CDCl_3$, 298 K) of **2**.

Synthesis of 4-(5,5-difluoro-2,8-diiodo-1,3,7,9-tetramethyl-5*H*-4λ[4],5λ[4]-dipyrrolo[1,2-c:2',1'-f][1,3,2]diazaborinin-10-yl)benzaldehyde (4)

4-(5,5-difluoro-1,3,7,9-tetramethyl-5*H*-4λ[4],5λ[4]-dipyrrolo[1,2-c:2',1'-f][1,3,2]diazaborinin-10-yl)benzaldehyde (**3**, 0.18 g, 0.49 mmol, 1 eq), NIS (0.34 g, 1.48 mmol, 3 eq) were dissolved in DCM (15 mL) and stirred for additional 12 h at room temperature. The mixture was concentrated and the crude product purified by silica gel column chromatography using a gradient from n-pentane/DCM (1/1, (*v*/*v*)) to DCM as eluent.
Yield: 0.28 g (**4**, 0.47 mmol, 96%) of a dark red solid.

<u>Characterization of **4**</u>:

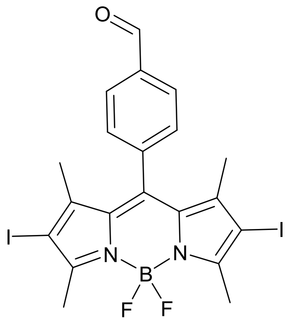

Chemical Formula: $C_{20}H_{17}BF_2I_2N_2O$
Exact Mass: 603.9491
Molecular Weight: 603.9847

^{1}H NMR (400 MHz, $CDCl_3$): δ (in ppm) = 10.15 (s, 1H-ALD), 8.08 (d, *J* = 8.2 Hz, 2H-Ar), 7.51 (d, *J* = 8.1 Hz, 2H-Ar), 2.67 (s, 6H-BODIPY), 1.39 (s, 6H-BODIPY).
^{13}C NMR (101 MHz, $CDCl_3$): δ (in ppm) = 191.3, 157.6, 145.0, 141.0, 139.3, 137.0, 130.6, 129.0, 86.2, 86.2, 86.1, 17.2, 16.2.
ESI-MS (TOF)**:** *m*/*z* 626.93882 $[M+Na]^+$, calculated for $C_{20}H_{17}N_2OBF_2I_2Na$: 626.93872

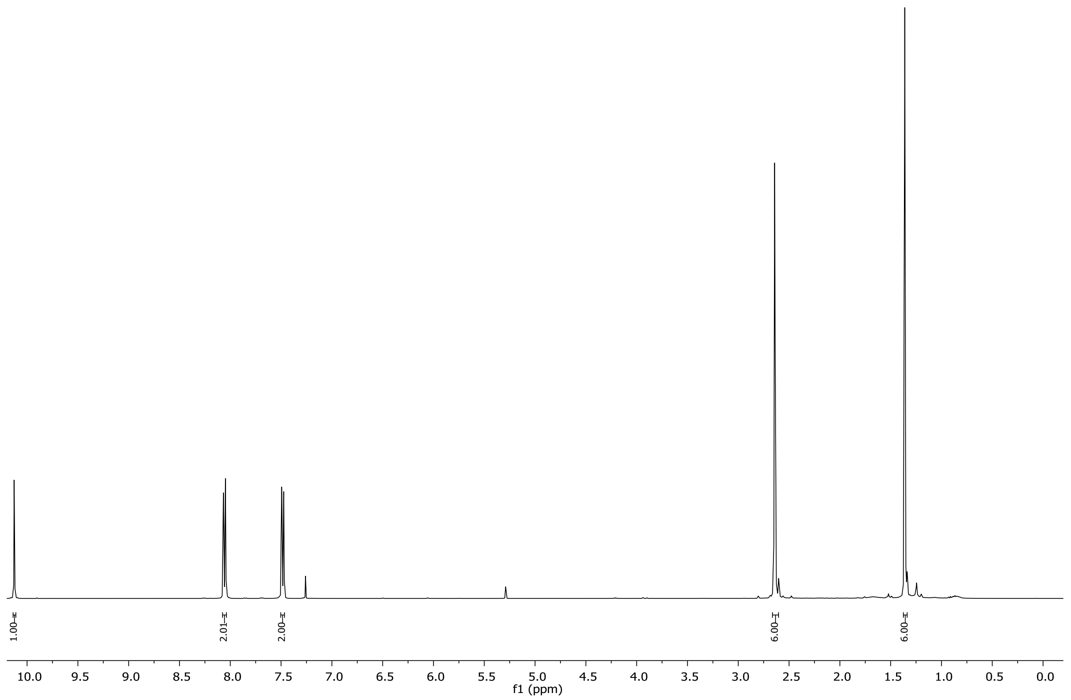

Figure S5. ^{1}H NMR (400 MHz, $CDCl_3$, 298 K) of **4**.

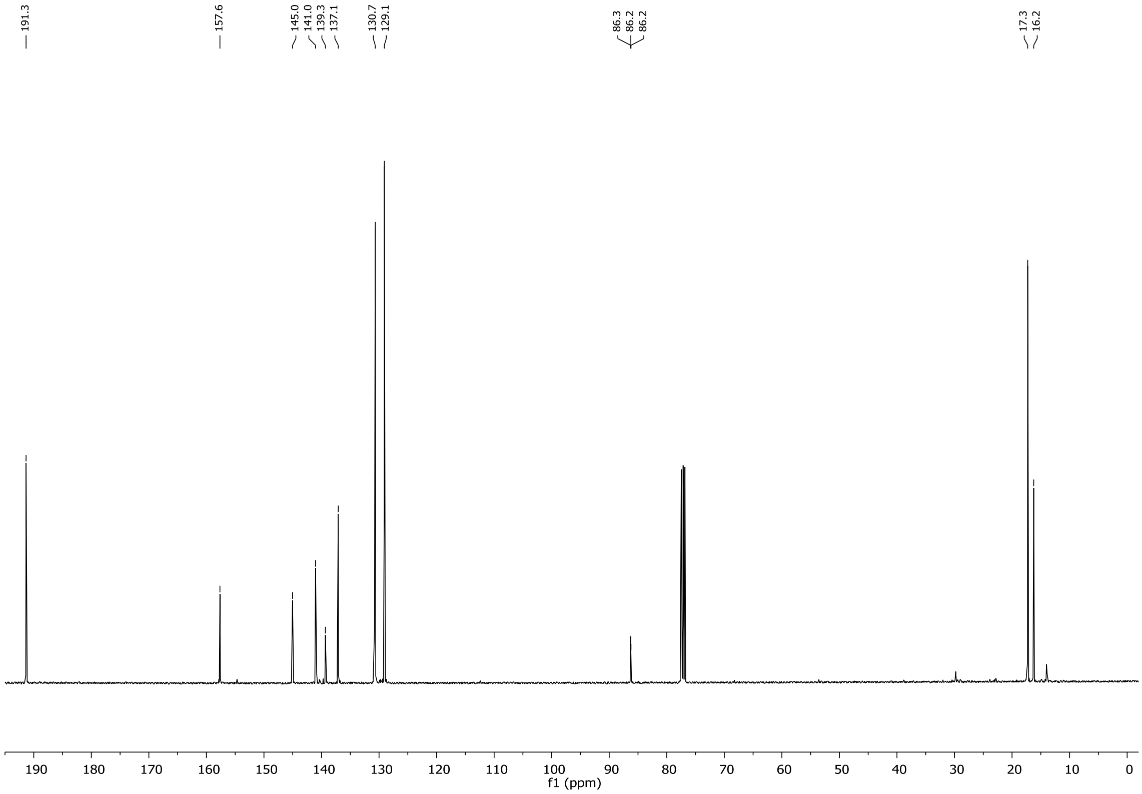

Figure S6. ^{13}C NMR (101 MHz, $CDCl_3$, 298 K) of compound **4**.

Synthesis of 4-(5,5-difluoro-2-iodo-1,3,7,9-tetramethyl-8-((3,4,5-tris(2-(2-(2-methoxyethoxy)ethoxy)ethoxy)phenyl)ethynyl)-5*H*-5λ[4],6λ[4]-dipyrrolo[1,2-c:2',1'-f][1,3,2]diazaborinin-10-yl)benzaldehyde (6)

4-(5,5-difluoro-2,8-diiodo-1,3,7,9-tetramethyl-5*H*-4λ[4],5λ[4]-dipyrrolo[1,2-c:2',1'-f][1,3,2]diazaborinin-10-yl)benzaldehyde (**4**, 2.36 g, 3.90 mmol, 1 eq), $(PPh_3)_4Pd$ (0.23 g, 0.20 mmol, 0.05 eq) and CuI (37.1 mg, 0.20 mmol, 0.05 eq) were dissolved in a mixture of anhydrous THF (10 mL) and NEt_3 (10 mL) and subjected to three vacuum-argon cycles The reaction mixture was heated to 50 °C and 5-ethynyl-1,2,3-tris(2-(2-(2-methoxyethoxy)ethoxy)ethoxy)benzene (**5,** 1.84 g, 3.11 mmol, 0.8 eq) dissolved in THF (100 mL) was added dropwise over the course of 2 hours to the solution and stirred for additional 12 h at 50°C. The solvent was removed and the crude product purified by silica gel column chromatography using a gradient from DCM to DCM/MeOH (5 %) as eluent, followed by reverse phase (C18) column chromatography using a gradient from water/ACN (9/1, (*v*/*v*)) to ACN as eluent.
Yield: 0.90 g (**6**, 1.21 mmol, 39%) of a black solid.

Characterization of **6**:

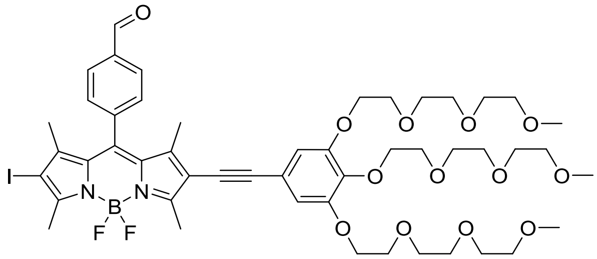

Chemical Formula: $C_{49}H_{64}BF_2IN_2O_{13}$
Exact Mass: 1064.3514
Molecular Weight: 1064.7633

^{1}H NMR (400 MHz, $CDCl_3$): δ (in ppm) = 10.08 (s, 1H-ALD), 8.01 (d, *J* = 8.2 Hz, 2H-Ar), 7.44 (d, *J* = 8.1 Hz, 2H-Ar), 6.61 (s, 2H-Ar-TEG), 4.24 – 4.02 (m, 6H-TEG), 3.89 – 3.44 (m, 30H-TEG), 3.30 (m, 9H-TEG), 2.62 (m, 6H-BODIPY), 1.36 (m, 6H-BODIPY).
^{13}C NMR (101 MHz, $CDCl_3$): δ (in ppm) = 191.3, 159.3, 157.3, 152.5, 144.4, 143.9, 140.8, 139.7, 139.4, 137.0, 130.5, 129.0, 117.9, 111.1, 96.9, 80.2, 72.4, 71.9, 71.9, 70.8, 70.7, 70.7, 70.6, 70.5, 70.5, 69.6, 68.9, 59.0, 59.0, 34.1, 29.7, 22.3, 17.1, 14.1, 13.7.
ESI-MS (TOF)**:** *m*/*z* 1087.34154 $[M+Na]^+$, calculated for $C_{49}H_{64}N_2O_{13}BF_2INa$: 1087.34148

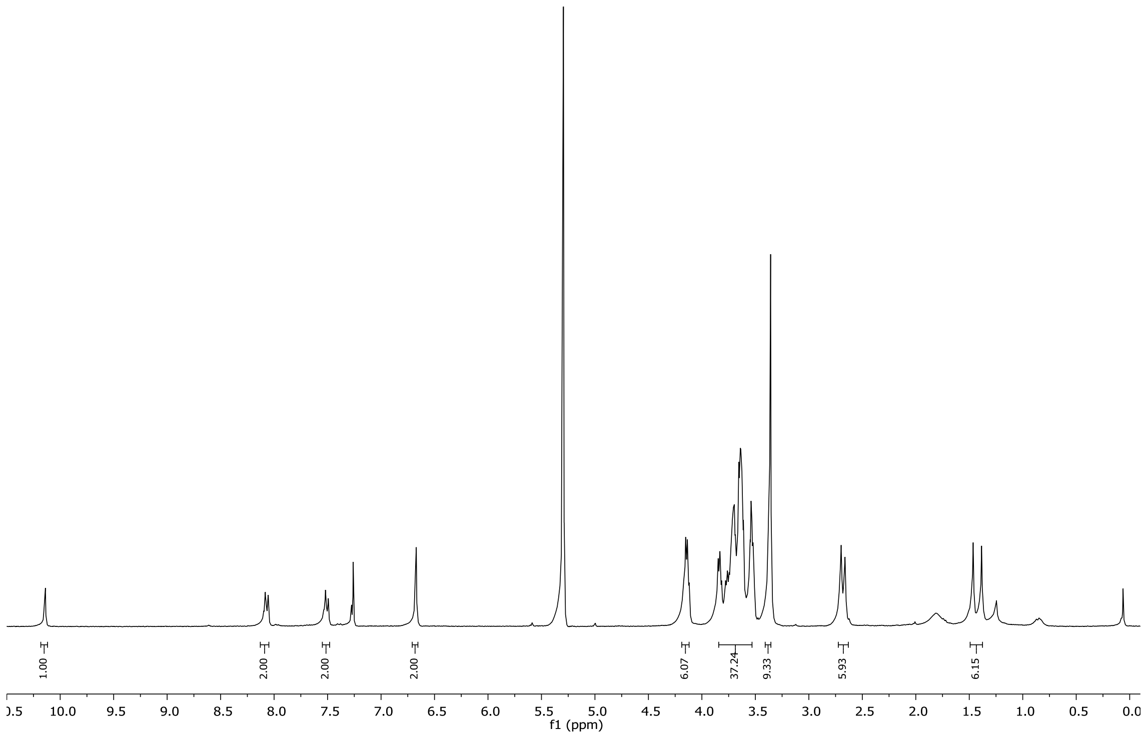

Figure S7. ^{1}H NMR (400 MHz, $CDCl_3$, 298 K) of **6**.

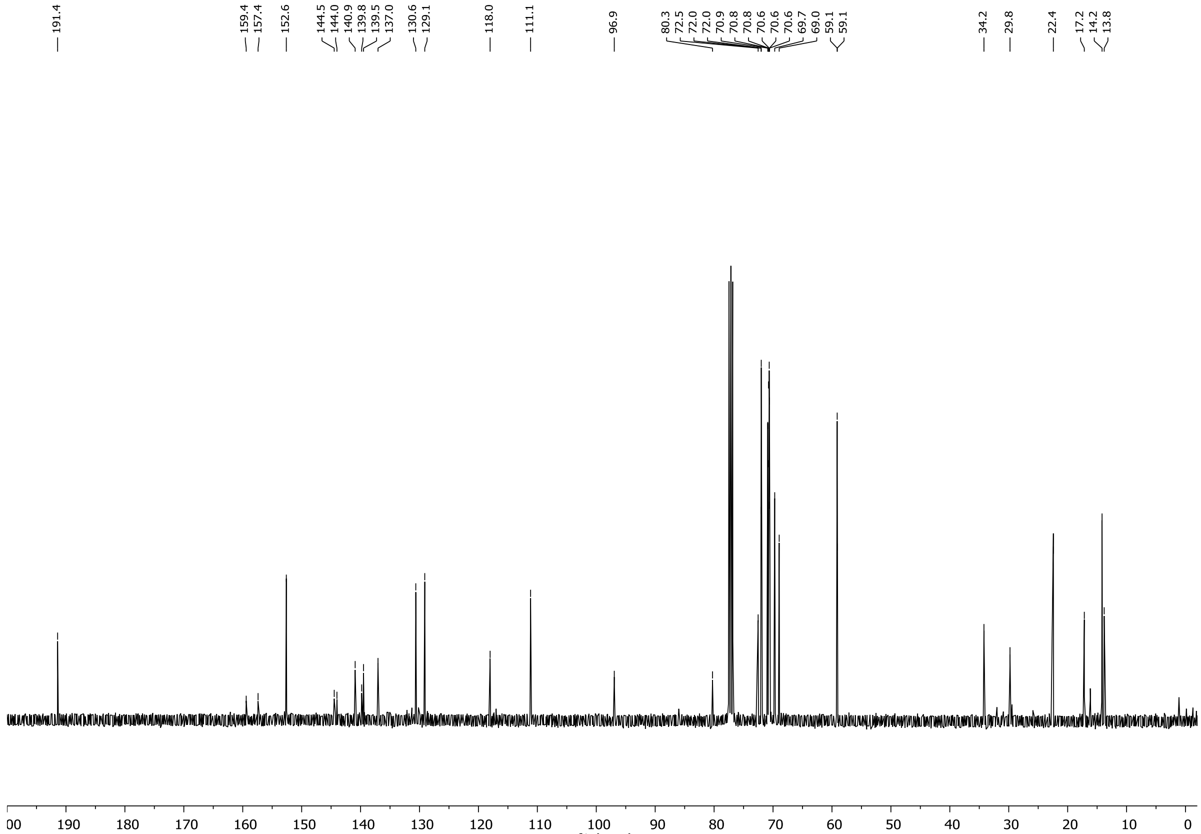

Figure S8. ^{13}C NMR (101 MHz, $CDCl_3$, 298 K) of compound **6**.

Synthesis of 4-((5,5-difluoro-10-(4-formylphenyl)-1,3,7,9-tetramethyl-8-((3,4,5-tris(2-(2-(2-methoxyethoxy)ethoxy)ethoxy)phenyl)ethynyl)-5*H*-5λ[4],6λ[4]-dipyrrolo[1,2-c:2',1'-f][1,3,2]diazaborinin-2-yl)ethynyl)benzoic acid (1)

4-(5,5-difluoro-2-iodo-1,3,7,9-tetramethyl-8-((3,4,5-tris(2-(2-(2-methoxyethoxy)ethoxy)ethoxy)phenyl)ethynyl)-5*H*-5λ[4],6λ[4]-dipyrrolo[1,2-c:2',1'-f][1,3,2]diazaborinin-10-yl)benzaldehyde (**6**, 912 mg, 0.86 mmol, 1 eq), $(PPh_3)_4Pd$ (49.5 mg, 42.8 µmol, 0.05 eq) and CuI (8.2 mg, 42.8 µmol, 0.05 eq) were dissolved in a mixture of anhydrous THF (6 mL) and NEt_3 (2 mL) and subjected to three vacuum-argon cycles. The reaction mixture was heated to 50 °C and 4-ethynylbenzoic acid (**7,** 188.1 mg, 1.29 mmol, 1.5 eq) was added to the solution and stirred for additional 12 h at 50°C. All solvents were removed and the crude product was purified by silica gel column chromatography using a gradient from DCM to DCM/MeOH (20 %) as eluent, followed by reverse phase (C18) column chromatography using a gradient from water/ACN (9/1, (*v*/*v*)) to ACN as eluent.
Yield = 866 mg (**1**, 0.80 mmol, 93%) of a blue solid.

Characterization of **1**:

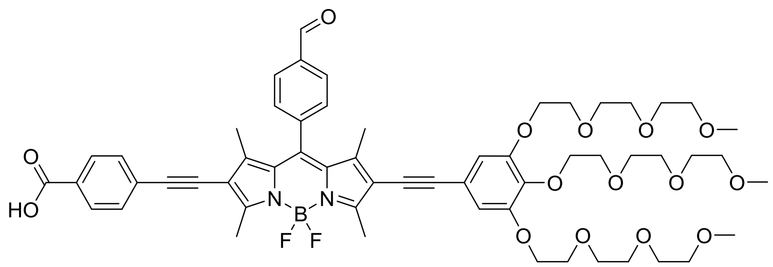

Chemical Formula: $C_{58}H_{69}BF_2N_2O_{15}$
Exact Mass: 1082.4759
Molecular Weight: 1082.9958

^{1}H NMR (400 MHz, $CDCl_3$): δ (in ppm) = 10.15 (s, 1H-ALD), 8.08 (d, *J* = 7.9 Hz, 2H-Ar), 7.99 (d, *J* = 8.1 Hz, 2H-Ar), 7.55 (d, *J* = 7.8 Hz, 2H-Ar), 7.46 (d, *J* = 8.0 Hz, 2H-Ar), 6.68 (s, 2H-Ar-TEG), 4.15 (m, 6H-TEG), 3.99 – 3.60 (m, 24H-TEG), 3.53 (m, 6H-TEG), 3.36 (m, 9H-TEG), 2.71 (m, 6H-BODIPY), 1.49 (s, 3H-BODIPY), 1.42 (s, 3H-BODIPY).
^{13}C NMR (101 MHz, $CDCl_3$): δ (in ppm) = 191.5, 169.1, 159.9, 158.8, 152.6, 143.9, 143.4, 140.8, 140.5, 139.5, 137.1, 131.1, 130.7, 130.1, 129.2, 129.0, 128.4, 118.1, 117.2, 115.8, 111.3, 109.1, 97.1, 96.4, 85.0, 80.2, 72.6, 72.1, 72.0, 70.9, 70.8, 70.7, 70.6, 70.6, 69.8, 68.9, 59.1, 14.0, 13.9, 13.8, 13.7.
ESI-MS (TOF)**:** *m*/*z* 1105.46610 $[M+Na]^+$, calculated for $C_{58}H_{69}N_2O_{15}BF_2$ Na: 1105.46609

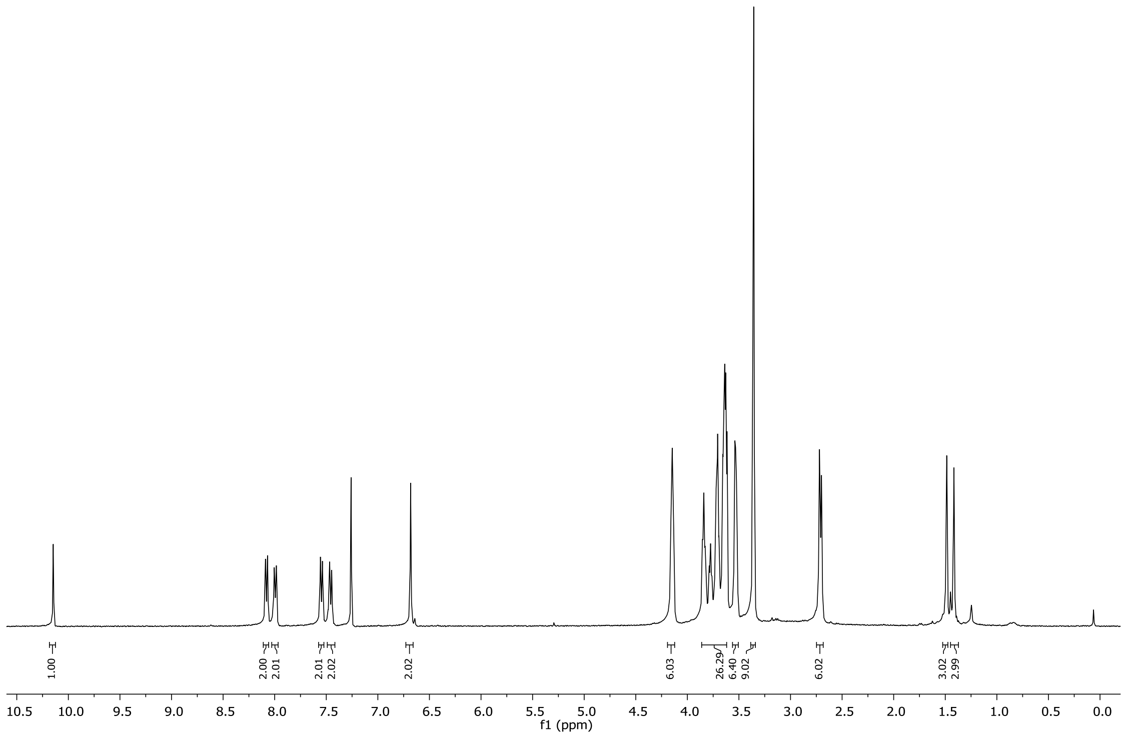

Figure S9. ^{1}H NMR (400 MHz, $CDCl_3$, 298 K) of compound **1**.

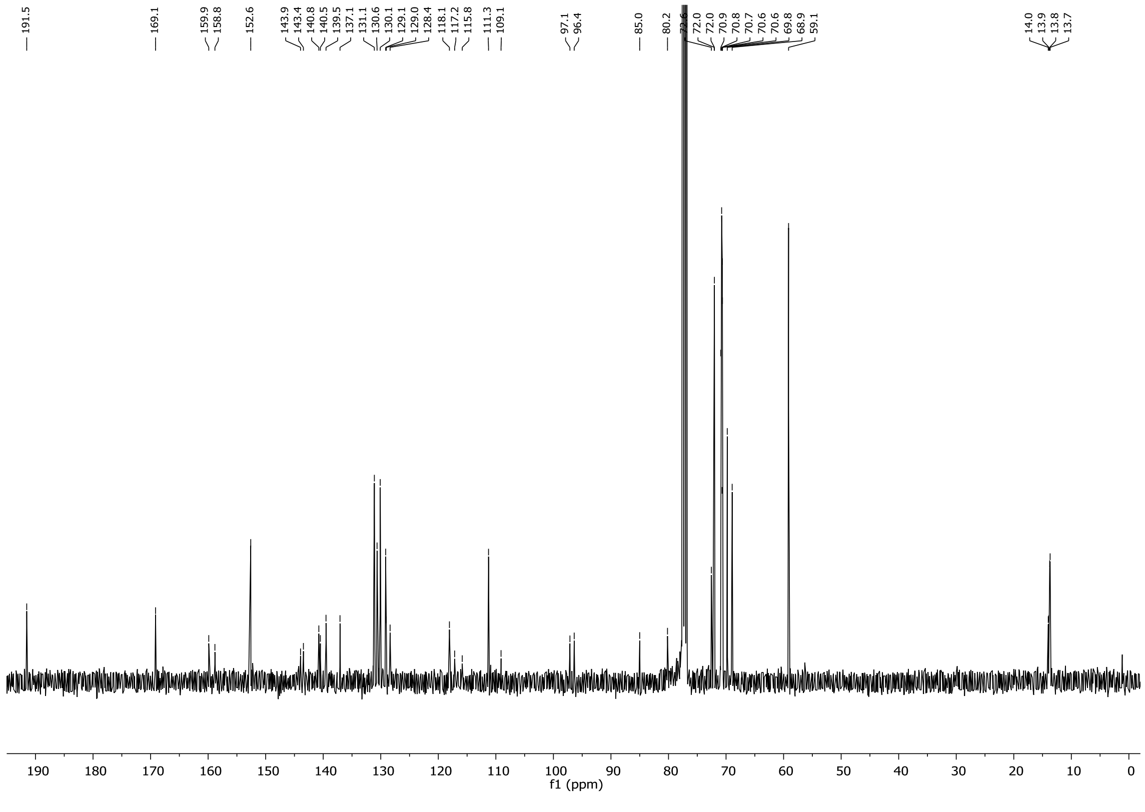

Figure S10. ^{13}C NMR (101 MHz, $CDCl_3$, 298 K) of compound **1**.

Results and Discussion

Nucleation-Elongation model for Cooperative Supramolecular Polymerizations

The equilibrium between the monomeric and supramolecular species can be described in a cooperative process with the *Nucleation-Elongation model* which is developed by Ten Eikelder, Markvoort and Meijer [7-8] This model is used to describe the aggregation of **B** which exhibits a non-sigmoidal cooling curve as shown in fluorescence and uv-vis temperature-dependent experiments. The model extends nucleation-elongation based equilibrium models for growth of supramolecular homopolymers to the case of two monomer and aggregate types and can be applied to symmetric supramolecular copolymerizations, as well as to the more general case of nonsymmetric supramolecular copolymerizations.
In a cooperative process, the polymerization occurs by a nucleation step, to a nucleus size assumed of B, and a following elongation step. The values T_e, $\Delta H°_{nucl}$, $\Delta H°$ and $\Delta S°$ can be determined by a non-linear least-square analysis of the experimental melting curves. The equilibrium constants associated with the nucleation and elongation phases can be calculated using equations **1** and **2**:

Nucleation step: $K_n = e^{\left(\frac{-(\Delta H^o - \Delta H^o_{nucl}) - T\Delta S^o}{RT}\right)}$ $\quad (\mathbf{1})$

Elongation step: $K = e^{\left(\frac{-(\Delta H^o - T\Delta S^o)}{RT}\right)}$ $\quad (\mathbf{2})$

And the cooperativity factor (σ) is given by:

$$\sigma = \frac{K_n}{K_e} = e^{\left(\frac{\Delta H^o_{nucl}}{RT}\right)} \quad (\mathbf{3})$$

Denaturation model [9]

The denaturation model is based on the concentration-dependent supramolecular polymerization equilibrium model by Goldstein, [10] whereas the polymerization is described as a sequence of monomer addition equilibria.

$$[P_n] = K_n[P_{n-1}][X]$$

$$[P_{n+1}] = K_e[P_n][X]$$

$$[P_i] = K_e[P_{i-1}][X]$$

For cooperative model $K_n<K_e$ and for isodesmic process $K_n=K_e$. The concentration for each species P_i is given by $[P_i] = K_n^{i-1}[X]^i$ for $i \leq n$ and $[P_i] = K_e^{i-n}K_n^{n-1}[X]^i$ for $i > n$.

The dimensionless mass balance is obtained by inserting the dimensionless concentration $p_i = K_e[P_i]$, monomer concentration $x = K_e[X]$ and concentration of each species P_i (for $i \leq n$) : $p_i = \sigma^{i-1}x^i$ and for $i > n :\ p_i = \sigma^{n-1}x^i$):

$$x_{tot} = \sigma^{-1}\sum_{i=1}^{n} i(\sigma x)^i + \sigma^{n-i}\sum_{i=n+1}^{\infty} ix^i.$$

Both sums are evaluated by using standard expressions for converging series:

$$x_{tot} = \left(\frac{(\sigma x)^{n+1}(n\sigma x - n - 1)}{(\sigma x - 1)^2} + \frac{\sigma x}{(\sigma x - 1)^2}\right) - \sigma^{n-1}\left(\frac{x^{n+1}(nx - n - 1)}{(x - 1)^2}\right)$$

With $x_{tot} = c_{tot}K_e$ and c_{tot}: the total monomer concentration

The sum solved by standard numerical methods (Matlabfzerosolver) results the dimensionless monomer concentration x. Considering that every species with $i > 1$ is defined as aggregate, the degree of aggregation results in:

$$\varphi = \frac{x_{tot} - x}{x_{tot}}$$

Via $K_e = \exp(-\frac{\Delta G^0}{RT})$ the denaturation curves can be obtained with f defined as volume fraction of good solvent:

$$\Delta G^{0,} = \Delta G^0 + m\,f$$

It is assumed that the cooperativity factor σ is independent of the volume fraction and the m value involved in the elongation equals the m value involved in the nucleation.

The denaturation data need to be transformed into the normalized degree of aggregation, if fitted to the supramolecular polymerization equilibrium model:

$$(f) = \frac{A(f) - A(f = 0)}{A(f = 1) - A(f = 0)}$$

The optimization of the four needed parameters (ΔG_0, m, σ and p) to fit the equilibrium model to the experimental data (normalized degree vs f) is done by the non-linear least-squares analysis using Matlab (lsqnonlinsolver). The data is then fitted with the non-linear least squared regression (Levenberg-Marquardt algorithm).

Fluorescence Quantum Yields

The fluorescence quantum yields for **1** and **2** were calculated using Rhodamine 101 (MeOH) as standard (Φ_{ref} = 1.0) and using the following equation:

$$\Phi = \Phi_{ref} \frac{A_r\, I}{A\, I_f}$$

A: Absorption (set under 0.1) for reference and sample

I: Integral of emission-peak for reference and sample (for all samples an excitation wavelength λ_{em} = 530 nm was applied)

Table S1. Fluorescence Quantum Yields of **1** and **2** in different solvents

	Solvent	ϕ_F
**1**	DCM	53.4%
**2**	DCM	18.9%
**1**	H_2O	0.6%
**A**	H_2O	2.4%
**B**	H_2O	6.4%

Thermodynamic Parameters

The thermodynamic parameters for **1** and **2** (**Table S2-3**) were obtained by fitting the respective experimental data to the denaturation model[a] and nucleation-elongation model[b]. Thermodynamic parameters extracted by ITC dilution experiments were analysed with Nanoanalyse Data Analysis and fitted to the one binding site independent model. However, it has to be noticed that this model is not accurate for cooperative supramolecular polymers (**1** and **B)**, as it does not include the nucleation step. We made the approximation of selecting this fit because we were highly limited in the concentration range (low concentration led to insufficient heat signal whereas at high concentration only partial disassembly of supramolecular polymers occurs) and therefore were not able to monitor the whole monomer to aggregate transition. Nevertheless, this fit is a good approximation if we compare these thermodynamic parameters with those obtained by denaturation and VT UV-Vis studies.

Table S2. Thermodynamic parameters of supramolecular polymerization of **1**, **A** and **B**. [a]

c / µM	**1** ΔG_{298} / kJ mol^{-1}	**A** ΔG_{298} / kJ mol^{-1}	**B** ΔG_{298} / kJ mol^{-1}
8	-52.6	-47.6	-51.9
10	-51.9	-45.1	-53.5
20	-54.8	-43.8	-47.1
40	-50.1	-40.8	-44.9
∅	**-52.4**	**-44.3**	**-49.3**

Table S3. Thermodynamic parameters of supramolecular polymerization of **B** (THF/water (9/1)). [b]

	c / µM	ΔH_0 / kJ mol^{-1}	ΔS_0 / kJ mol^{-1} T^{-1}	ΔH_{nucl0} / kJ mol^{-1}	T_E / K	ΔG_{298} / kJ mol^{-1}	K_{el}	K_{nucl}	σ
**B** UV	8	-106.1	-0.24	-13.1	315.0	-31.7	120006.1	851.1	0.0068
**B** Emission	8	-108.9	-0.24	-16	322.3	-34.8	125013.5	322.2	0.0026

Table S4. Thermodynamic parameters of supramolecular polymerization of **1**, **A** and **B** extracted by ITC dilution experiments (298 K and THF/water (9/1)).

	c / mM	ΔH_{298} / kJ mol^{-1}	ΔS_0 / kJ mol^{-1} T^{-1}	ΔG_{298} / kJ mol^{-1}	$-T\Delta S_0$ / kJ mol^{-1}	$\frac{T\Delta S_0}{\Delta H_{298}}$ / a.u.
**1**	1	-286.1	-0.85	-30.4	255.7	0.91
**A**	1	-50.1	-0.09	-24.1	26.1	0.53
**B**	1	-110.7	-0.29	-25.8	84.9	0.77

Semiempirical calculations at the dispersion-corrected PM6 level in vacuum

Table S5. Dipole and energy of different Dimer/Tetramer of **1** and **2** (Figure S21-S23).

	Dipole / Debye	*Energy* / kJ/mol
**1_Tetramer**	8.611	-10809.24434
**2_Dimer 1**	11.949	-2011.09
**2_Dimer 2**	8.855	-1952.20
**2_Dimer 3**	4.612	-2025.87
**2_Dimer 4**	0.217	-2010.72
**2_Tetramer 3**	10.022	-4186.82219
**2_Tetramer 4**	1.055	-4156.13720

Supplementary Figures

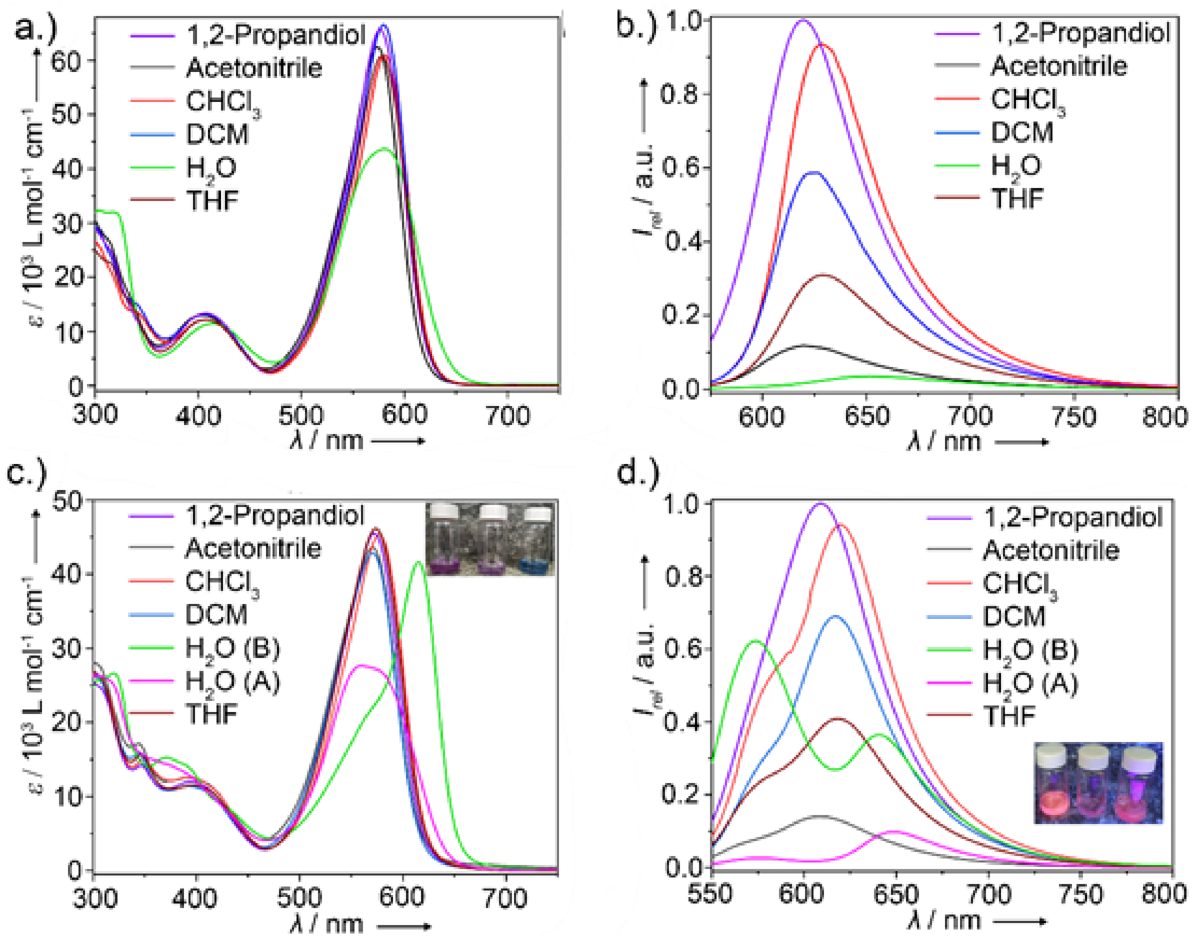

Figure S11. UV-Vis absorption (a, c) and emission spectra (b, d) of compounds **1** (top) and **2** (bottom) in different solvents (c = 20 µM) at 298 K. For emission spectra, an excitation wavelength of λ = 530 nm was applied. Insets (c, d): left monomer solution, middle **A**, right **B**.

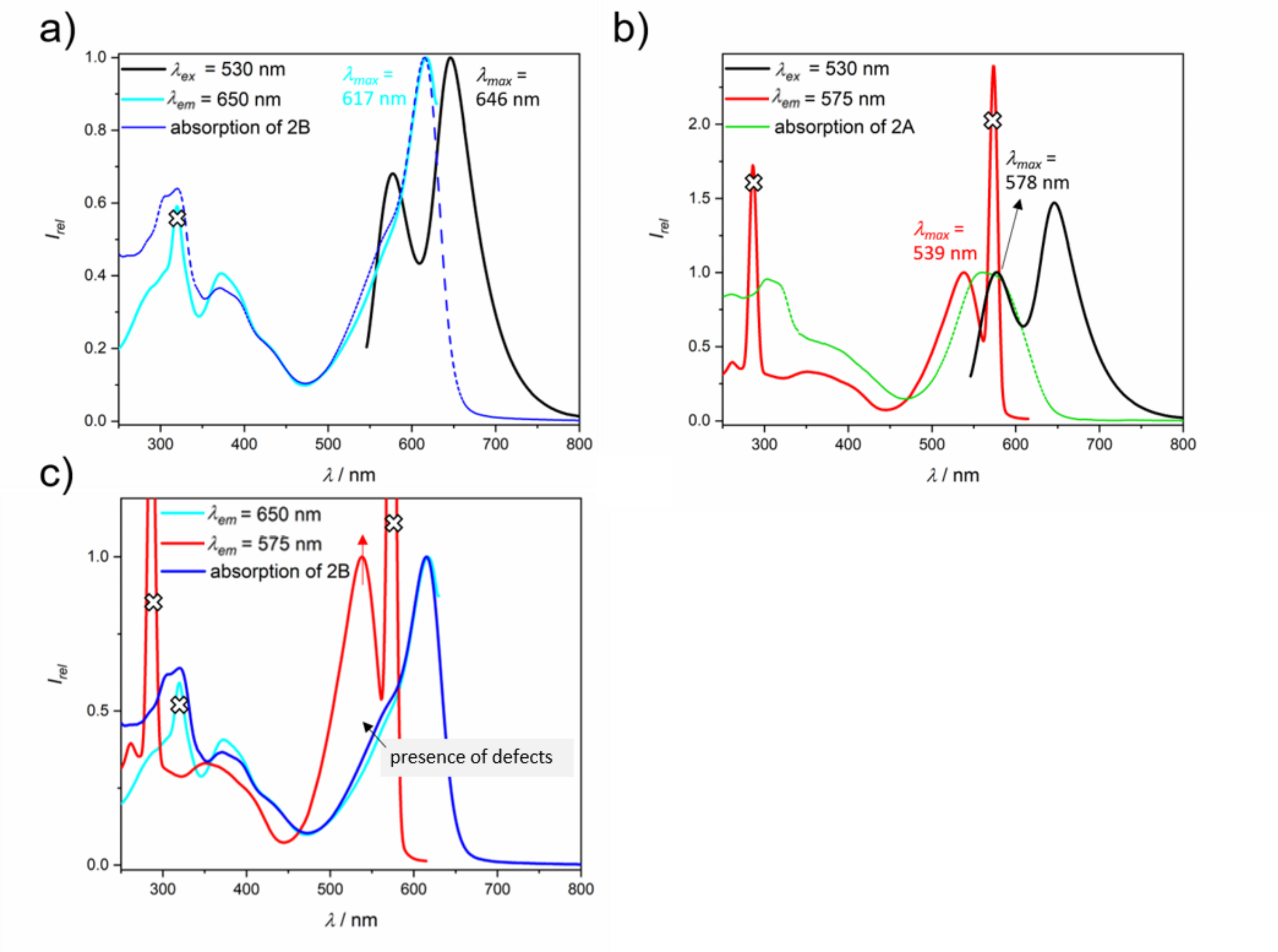

Figure S12. Emission-studies of **B** in H_2O (2 x 10^{-5} M, 298 K, λ_{ex} = 530 nm): a) excitation (λ_{em} = 650 nm) *vs.* absorption spectrum of **B**, b) excitation (λ_{em} = 575 nm) *vs.* absorption spectrum of **A**, c) both excitation spectra (λ_{em} = 575 and 650 nm) *vs.* absorption spectrum of **B**.

The emission spectrum of **B** exhibits two bands at 578 and 646 nm. To investigate whether this emission band at 578 nm is due to a rotational displacement of the BODIPY in the packing of **B** or another energetic state that coexists with the major packing of **B**, excitation spectra of both emission bands were measured. While the excitation of the emission band at 646 nm exhibits almost identical spectrum as the characteristic absorption spectrum (Figure S12a), the emission band at 578 nm exhibits a significantly different spectrum. Since this excitation spectrum does not merge with the absorption spectrum of **A**, this band is attributed to another energetic state, which coexists with the major J-type packing of **B** (Figure S12b). This is rationalized by the presence of some defects in the packing of **B,** as also shown by theoretical calculations (see scheme 2 and Figure 6). The degree of these defects is directly reflected in absorption spectrum by the intensity of the blue-shifted shoulder at 539 nm (Figure S12c).

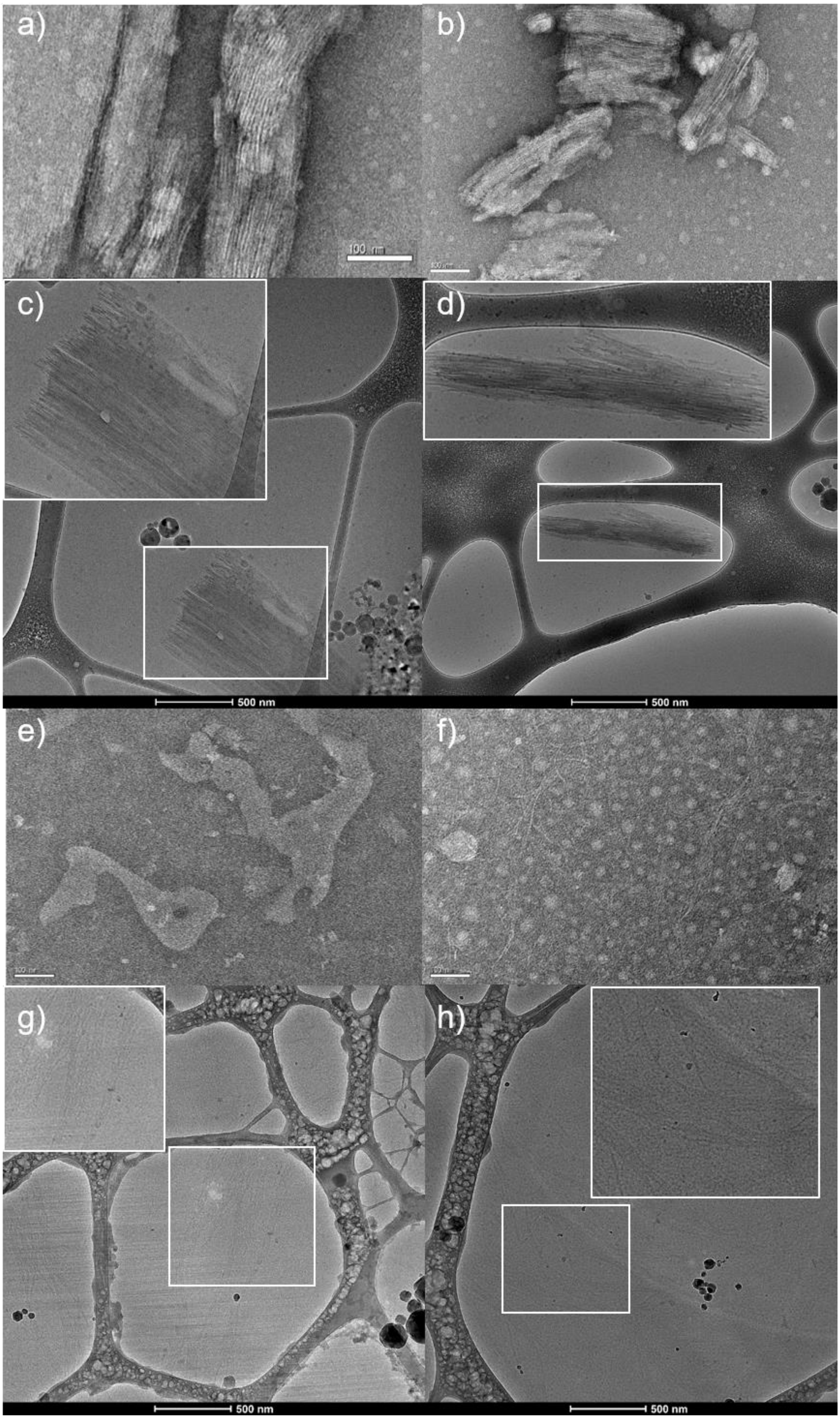

Figure S13. TEM images of **1** (a-b); **A** (e) and **B** (f) in H_2O (c = 2.0 x 10^{-5} M) drop-casted onto on a carbon-coated copper grid and stained with uranyl acetate. cryo-TEM images of **1** (c-d) and **B** (g-h) in H_2O (c = 2.0 x 10^{-5} M).

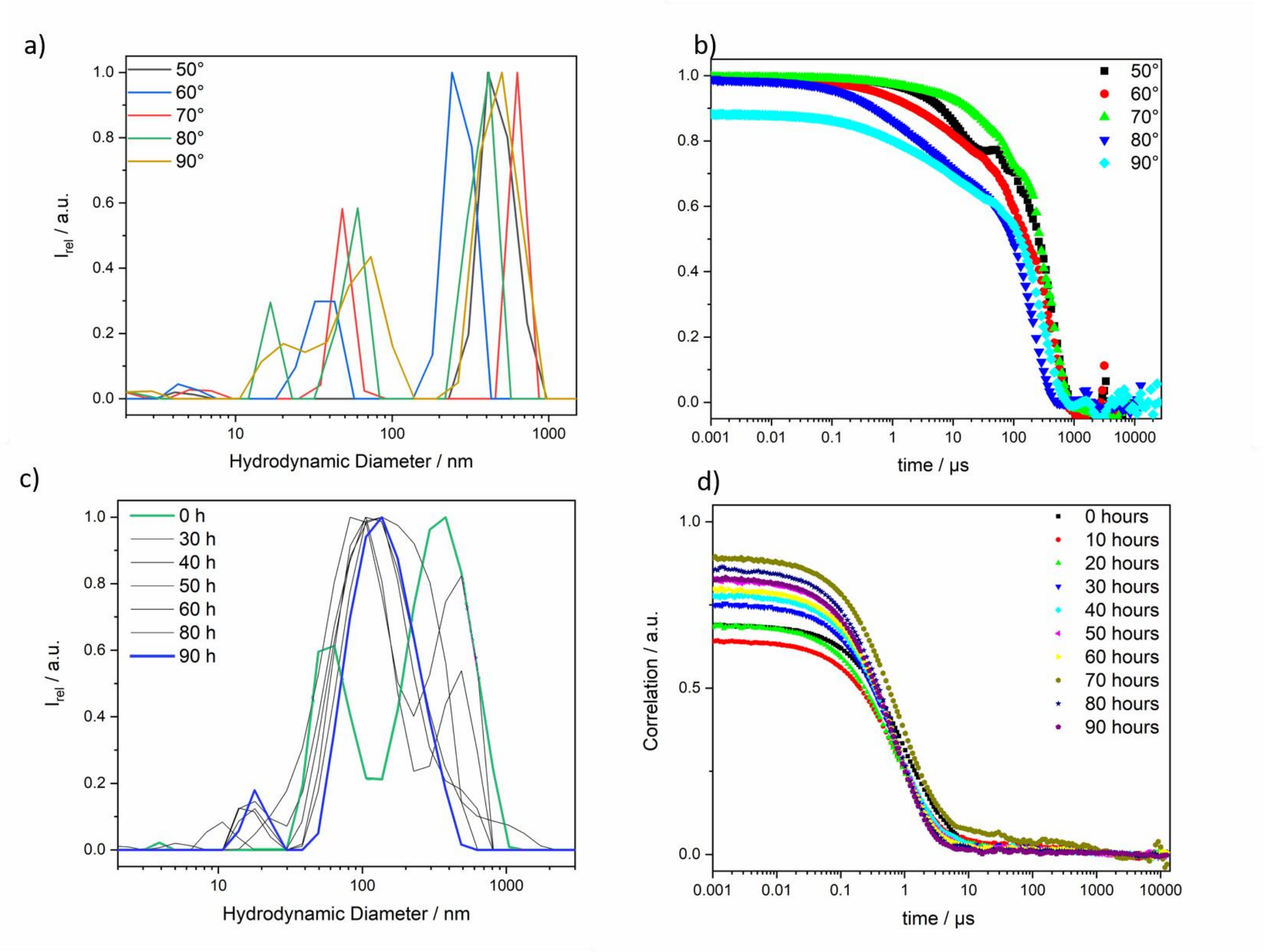

Figure S14. Angular-dependent DLS studies of **1** in H_2O (2 x 10^{-5} M, 298 K),: a) Size distribution and b) Correlation function. Time-dependent DLS (scattering angle 100°) studies of **2** in H_2O (2 x 10^{-5} M, 298 K): c) Size distribution and d) Correlation function.

For **1**, angular-dependent dynamic light scattering (DLS) studies reveal two size distributions (16-73 nm, 200-600 nm) that are highly dependent on the scattering angle, which underlines the anisotropy of these structures. The predominant species with larger hydrodynamic radii is expected to be the lamellar structures.

The transformation of **A** to **B** is also monitored by DLS: initially, two broad size distributions centered at 62 nm and 372 nm, which are additionally highly dependent on the angle, are observed. After 30 hours, the size distribution at lower hydrodynamic radii disappears, while the larger predominant species changes from 372 to 105 nm. After 50 hours, the species at around 400 nm disappears completely and no further changes in size distribution are observed.

*Since the laser of the DLS device operates at the wavelength of 633 nm, which overlaps with the absorption and emission of **B**, we cannot rule out the presence of artifacts that interfere with our results.

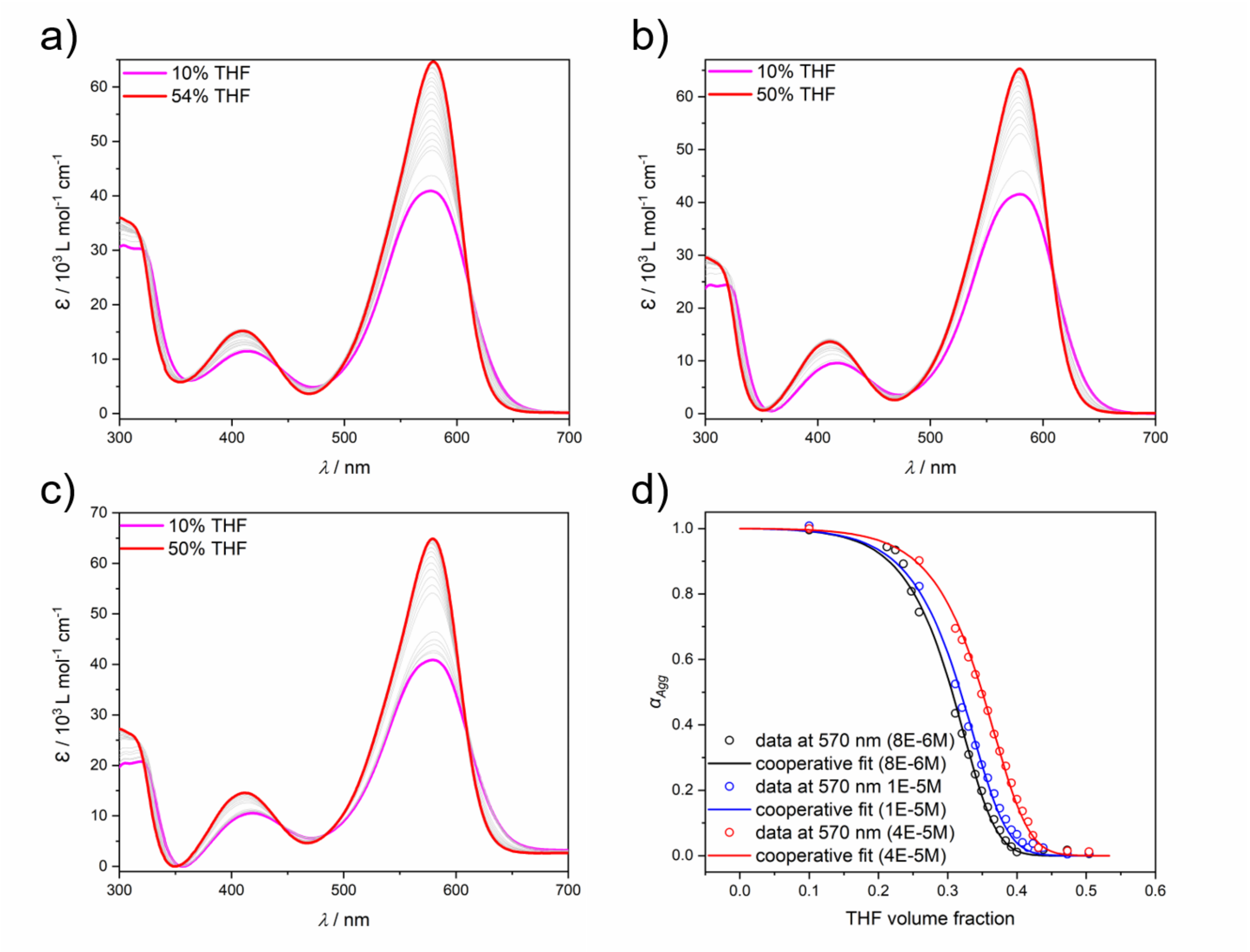

Figure S15. UV-Vis studies of **1** at different THF/water ratios, 298 K and a) c = 40 μM; b) 10 μM; c) 8 μM. d) Plot of α_{agg} at λ = 570 nm vs. THF volume fraction and fit to the denaturation model for different concentrations**.**

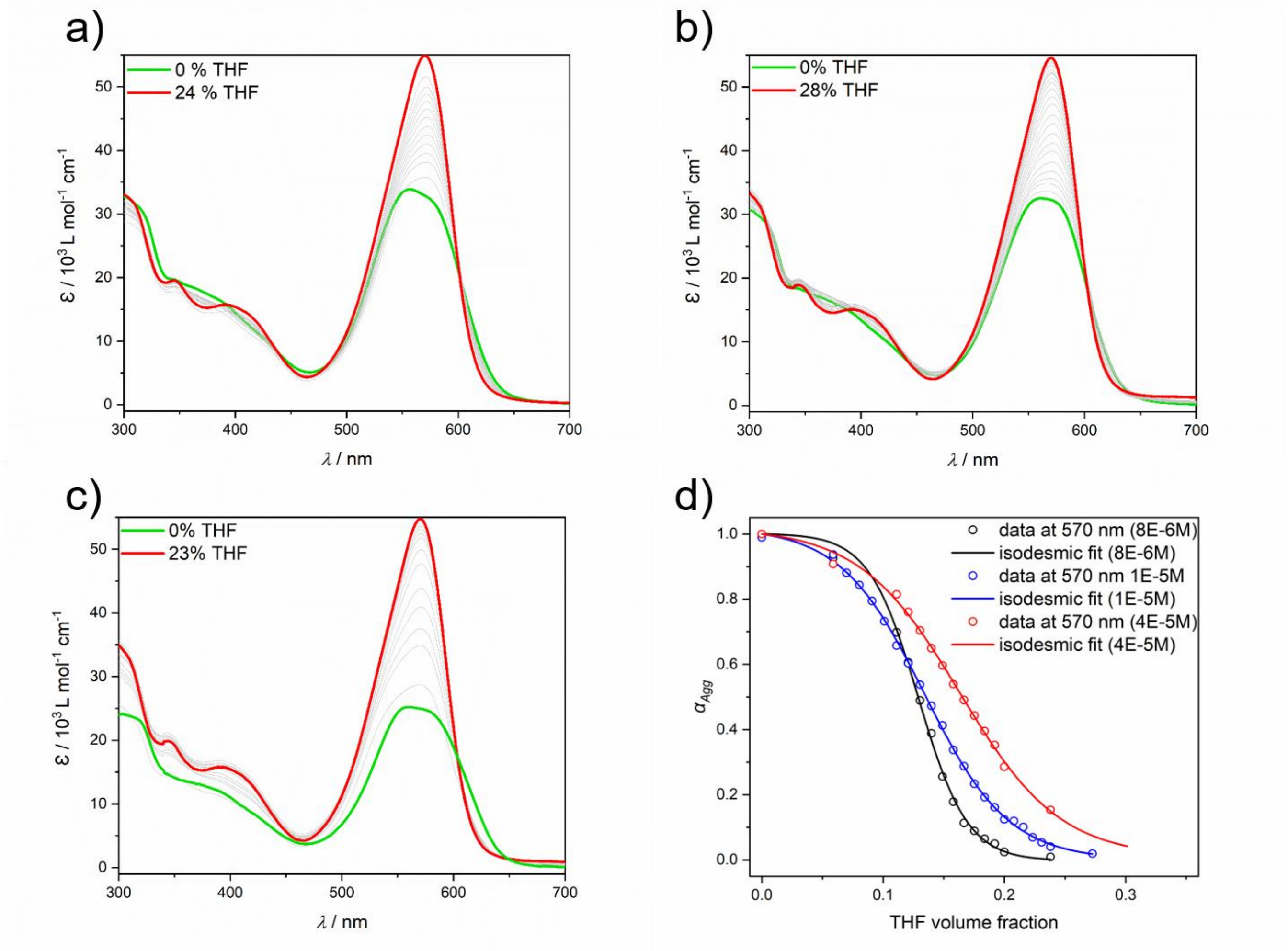

Figure S16. UV-Vis studies of **A** at different THF/water ratios, 298 K and a) c = 40 μM; b) 10 μM; c) 8 μM. d) Plot of α_{agg} at λ = 570 nm vs. THF volume fraction and fit to the denaturation model for different concentrations.

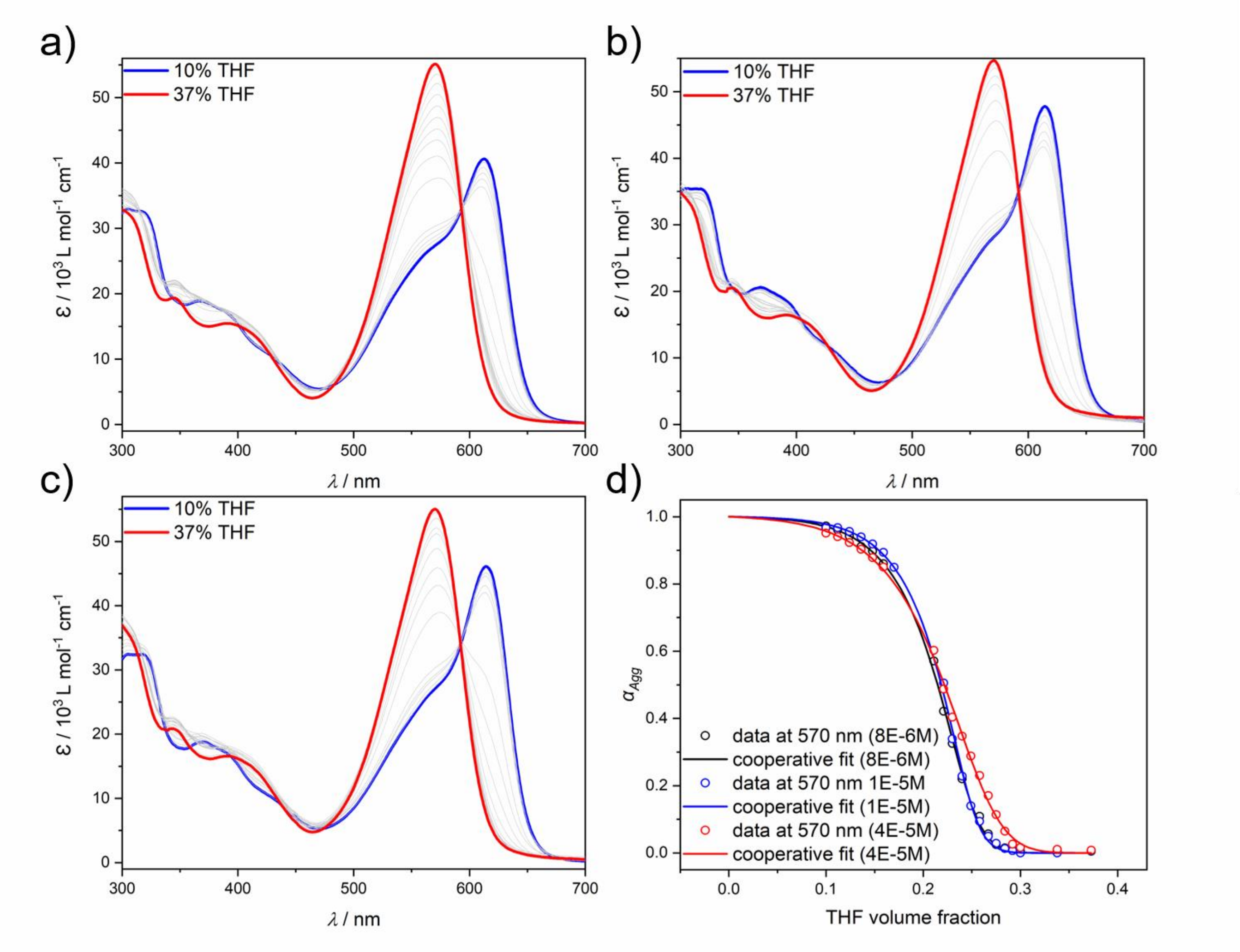

Figure S17. UV-Vis studies of **B** at different THF/water ratios, RT and a) c = 40 μM b) 10 μM c) 8 μM. d) Plot of α_{agg} at λ = 570 nm vs. THF volume fraction and fit to the denaturation model for different concentrations**.**

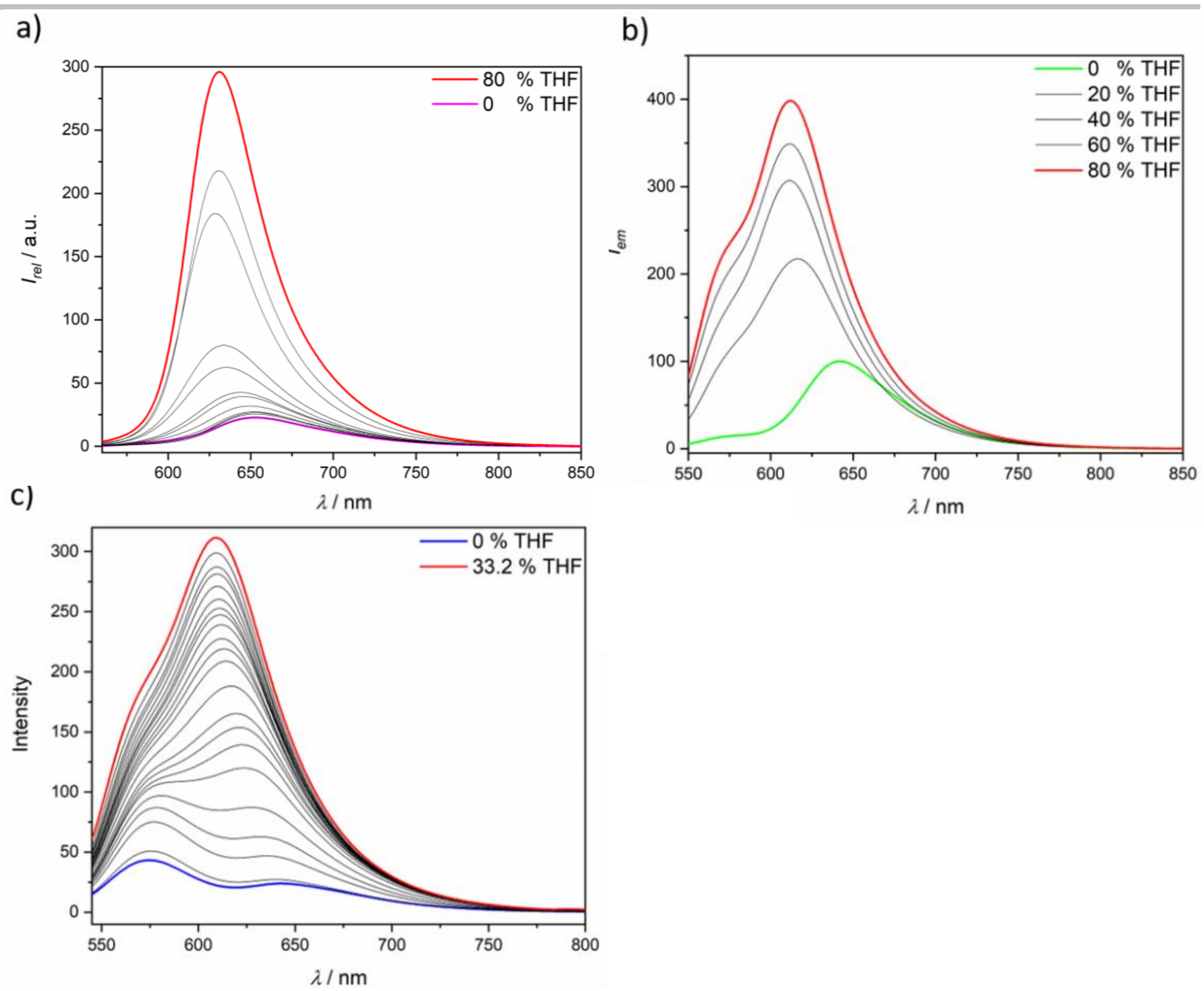

Figure S18. Emission studies of a) **1,** b) **A** and c) **B** at different THF/water ratios at *c* = 20 μM and 298 K.

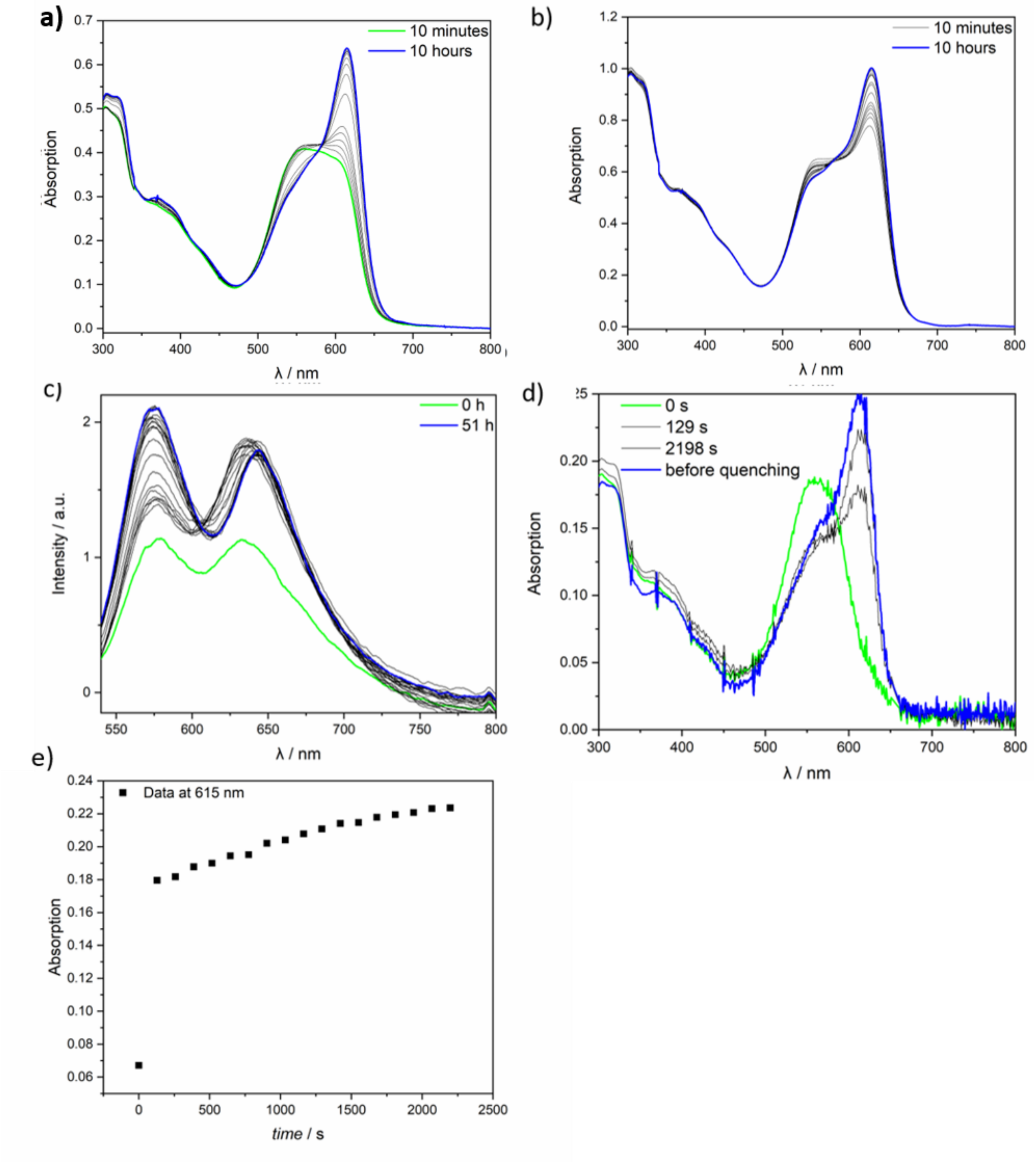

Figure S19. a) Time-dependent UV-vis studies monitoring the **A**→**B** transformation (2 x 10^{-5} M, 313.15 K). b) Time-dependent UV-vis studies monitoring the **A**→**B** transformation (4 x 10^{-5} M, 313.15 K). c) Time-dependent emission studies (2 x 10^{-5} M, 298 K). d) Thermal quenching and subsequent time dependent-studies of **2** monitored with UV-Vis: **B** was heated at 90 °C for 5 min, then quenched (rapid temperature drop) to 10 °C, and finally time-dependent UV-Vis changes were measured (8 x 10^{-6} M, 10% THF). e) Corresponding kinetic profile of thermal quenching experiment illustrated in figure d (8 x 10^{-6} M, 10% THF)

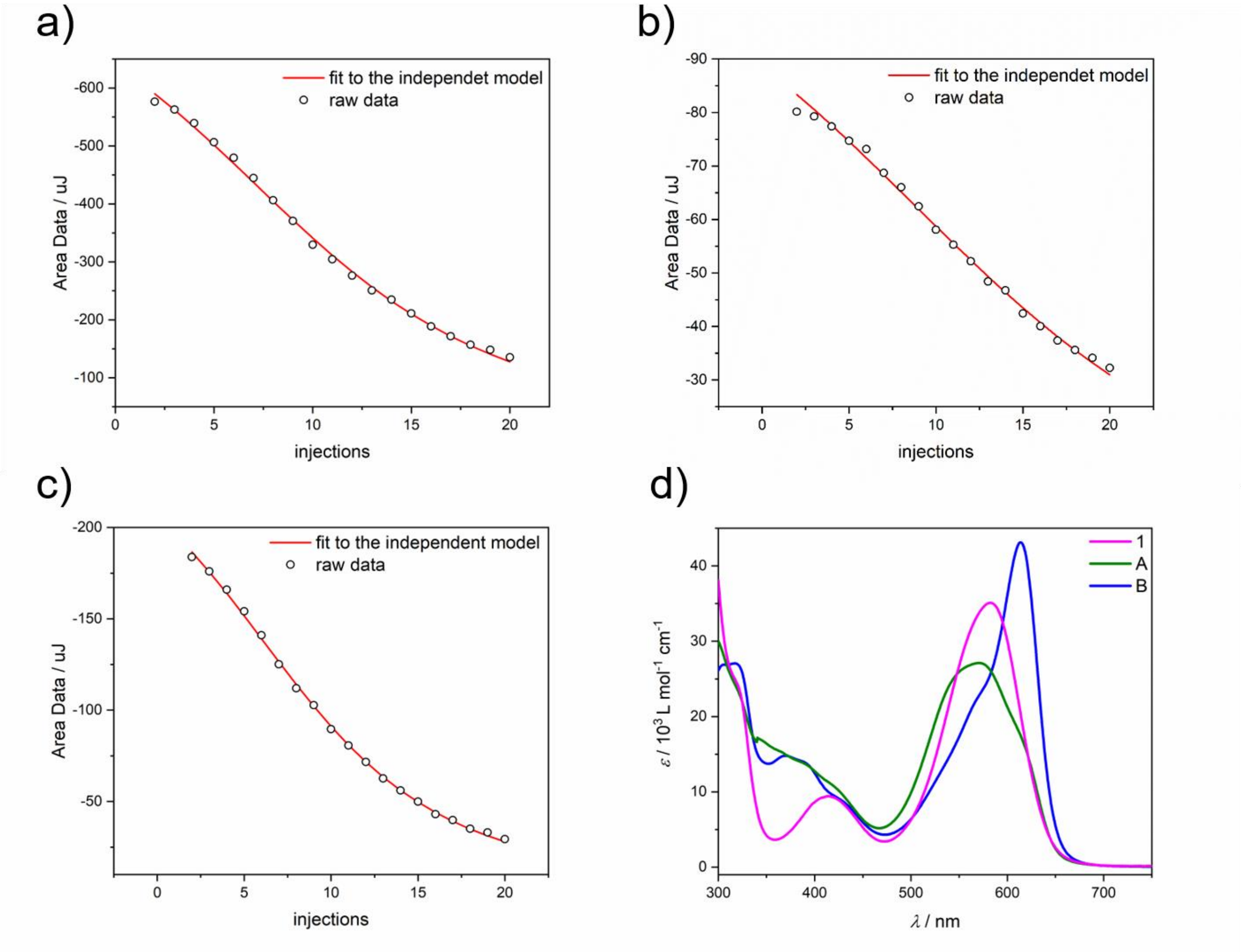

Figure S20. Independent fit of the area data of detected heat release obtained by ITC dilution experiments of **1** (a), **A** (b), **B** (c): injection of 1 mM water/THF (9/1) solution into pure water/THF (9/1) at 298 K. (d) UV-Vis of **1**, **A**, **B** (1 mM, water/THF (9/1), 298 K) measured after completion of the ITC dilution experiments. The spectral features of the three aggregates under the experimental conditions required for ITC studies (1mM) are nearly identical to those observed at lower concentrations in spectroscopic studies, which demonstrates the identity of the corresponding aggregates during the ITC measurements.

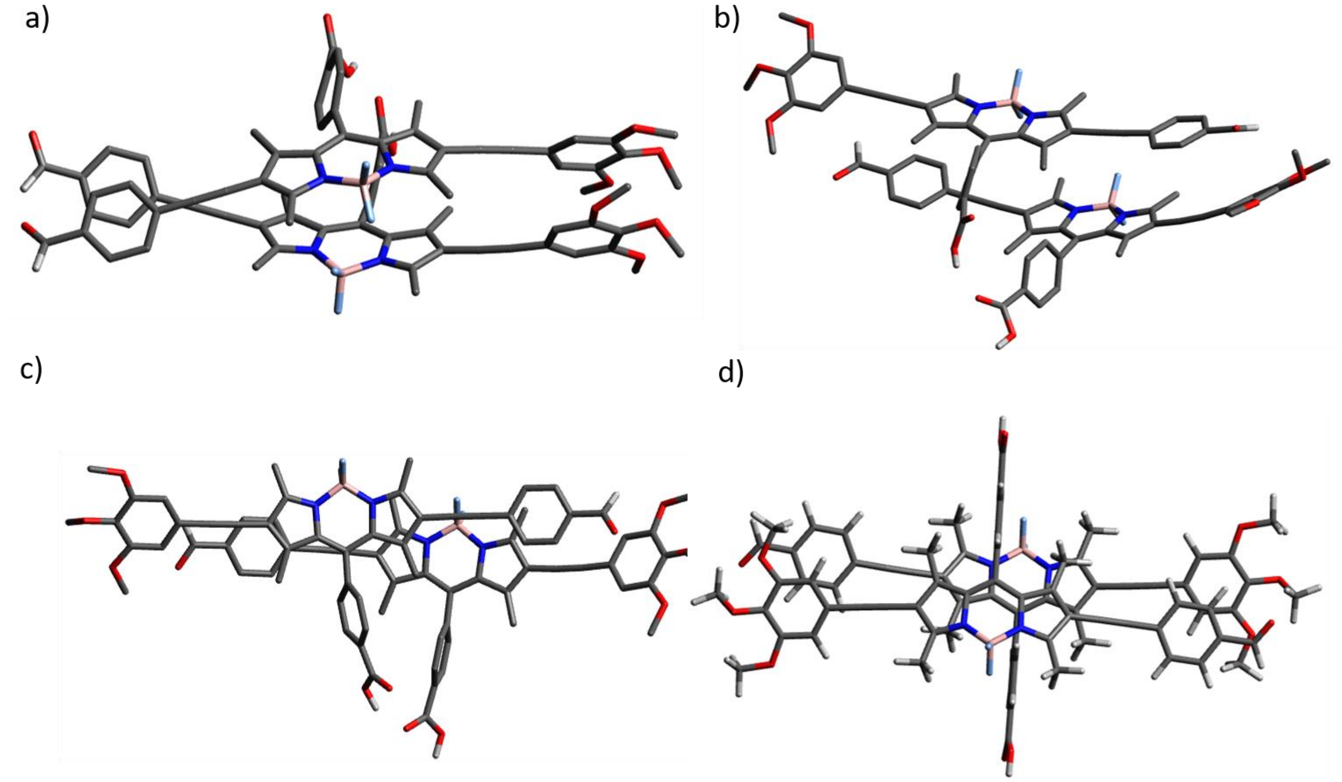

Figure S21. PM6-optimized dimers of **2** (TEG replaced by methoxy chains): a) 2_1, b) 2_3, c) 2_3, d) 2_4

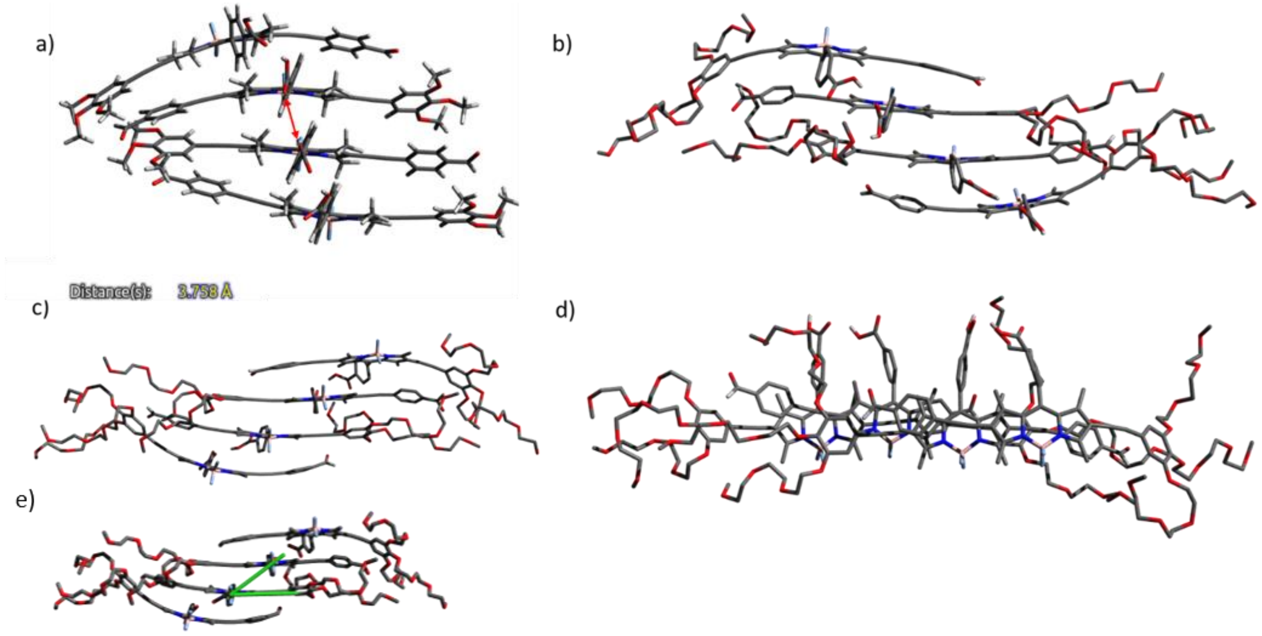

Figure S22. PM6-optimized tetramers of **2_3**: a) TEG replaced by methoxy chains, b,c,d,e) with TEG chains

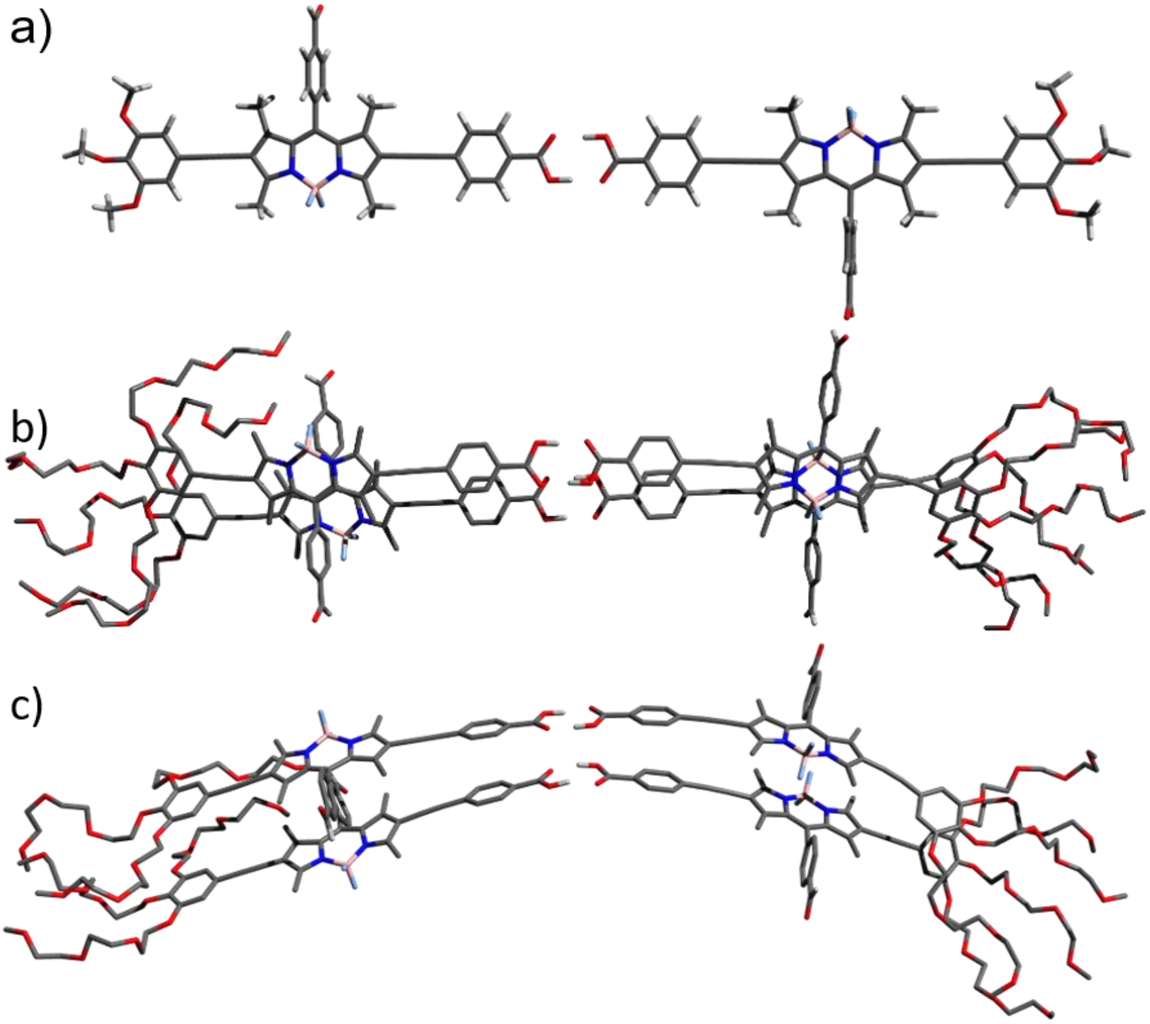

Figure S23. a) PM6-optimized Dimer (TEG replaced by methoxy chains), b,c) optimized Tetramer of **1**

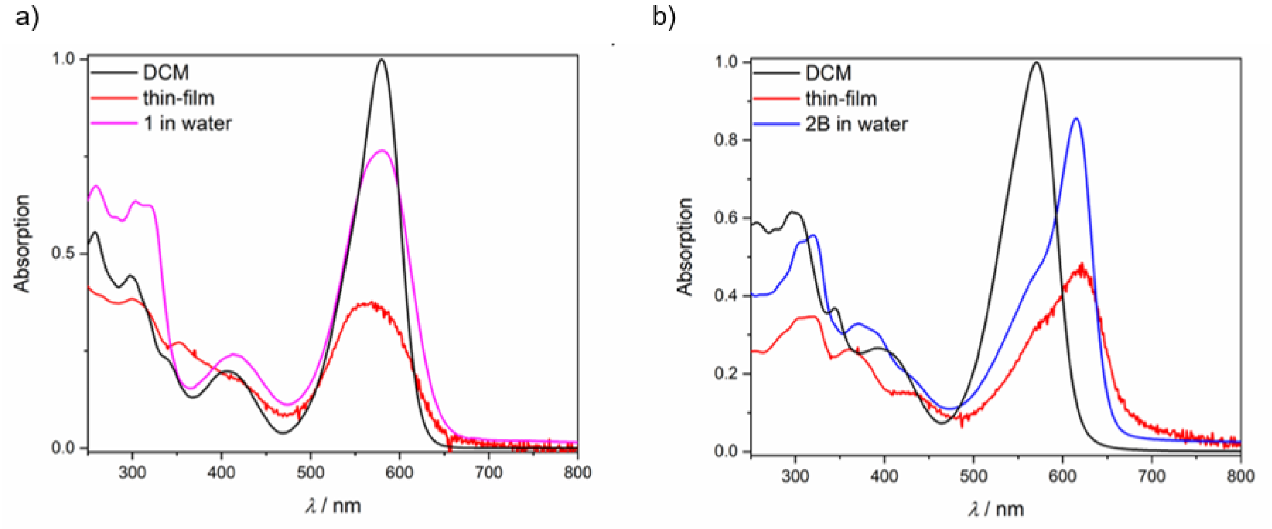

Figure S24. UV-Vis spectra of a) **1** and d) **2**, thin-film (black) vs. aggregates in water (red, *c* = 2 x 10^{-5} M).

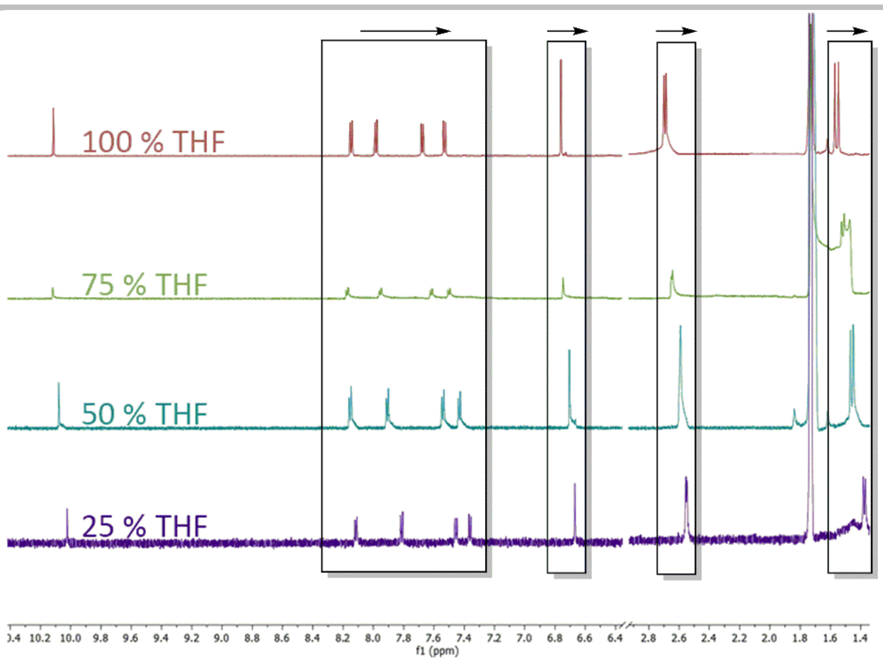

Figure S25. ^{1}H-NMR spectra of **1** at different ratios of water**/**THF.

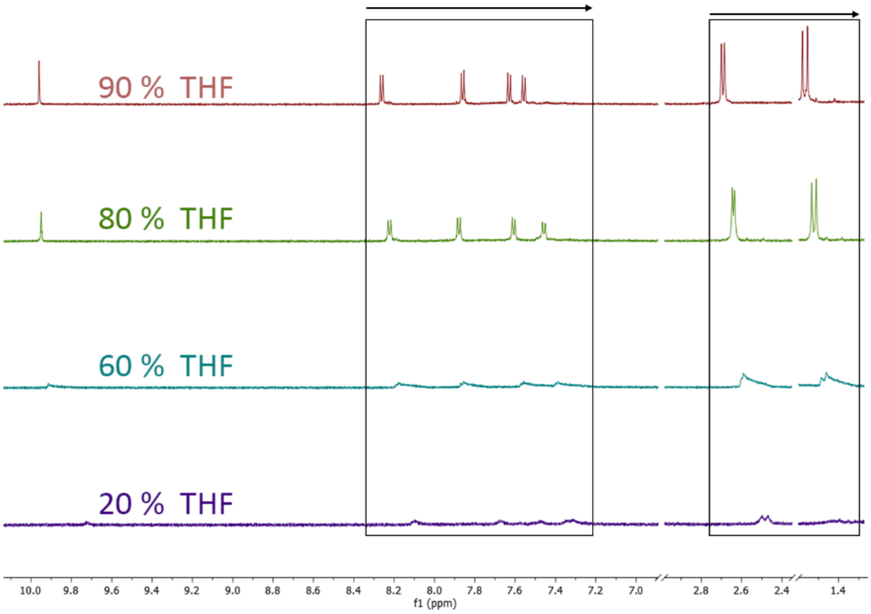

Figure S26. ^{1}H-NMR spectra of **2** at different ratios of water**/**THF.

References

[1] S. Kolemen, A. Bozdemir, Y. Cakmak, G. Barin, S. Erten-Ela, M. Marszalek, J. Yum, S. M. Zakeeruddin, M. K. Nazeeruddin, M. Gratzel, E. U. Akkaya *Chem. Sci.* **2011**, *2*, 949-954.
[2] J. W. Grate, G. C. Frye in *Sensors Update*, *Vol. 2* (Eds.: H. Baltes, W. Göpel, J. Hesse), Wiley-VCH, Weinheim, **1996**, pp. 10-20.
[3] Q. Li, Y. Yuea, Y. Guoa, S. Shaoa, *Sensors and Actuators B* **2012**, *173,* 797-801.
[4] F. A. Mandl,V. C. Kirsch,I. Ugur,E. Kunold, J. Vomacka,C. Fetzer,S. Schneider,K. Richter,T. M. Fuchs,I. Antes and S. A. Sieber, *Angew. Chem. Int. Ed.* **2016**, *55*, 14852-14857.
[5] M. Korzec, S. Kotowicz, K. Laba, M. Lapkowski, J. G. Malecki, K. Smolarek, S. Maćkowskian, E. Schab-Balcerzak, *Eur. J. Org. Chem.* **2018***,* 1756-1760.
[6] S. Chen, X. Huang, S. Decurtins, C. Albrecht, S. Liu, *Polyhedron* **2017**, *134*, 287-294.
[7] H. M. M. Ten Eikelder, A. J. Markwoort, T. F. A. De Greef, P. A. J. Hilbers, *J. Phys. Chem. B* **2012**, *116*, 5291-5301.
[8] A. J. Maarkvort, H. M. M. Ten Eikelder, P. J. J. Hilbers, T. F. A. De Greef, E. W. Meijer, *Nat. Commun.* **2011**, *2*, 509-517.
[9] P. A. Korevaar, C. Schaefer, T. F. A. de Greef, E. W. Meijer, *J. Am. Chem. Soc.* **2012**, *134*, 13482-13491.
[10] Goldstein, R. F.; Stryer, *L. Biophys. J.* **1986**, *50*, 583-599.
[11] I. Kaur, P. K. Dhiman, *IJOC* **2011**, *01*, 6.
[12] a) B. Matarranz, A. Sampedro, C. G. Daniliuc, G. Fernández, *Crystals* **2018**, *8*, 436; b) M. Takasuka, K. Matsumura, N. Ishizuka, *Vibrational Spectroscopy* **2001**, *25*, 63.
